# Hierarchically MOF‐Based Porous Monolith Composites for Atmospheric Water Harvesting

**DOI:** 10.1002/adma.202413353

**Published:** 2025-07-02

**Authors:** Mahyar Panahi‐Sarmad, Tianyu Guo, Seyyed Alireza Hashemi, Ahmadreza Ghaffarkhah, Stefan Wuttke, Mohammad Arjmand, Orlando J. Rojas, Feng Jiang

**Affiliations:** ^1^ Department of Wood Science Faculty of Forestry The University of British Columbia 2036 Main Mall Vancouver BC V6T 1Z4 Canada; ^2^ Bioproducts Institute University of British Columbia 2385 Agronomy Rd & East Mall Vancouver BC V6T 1Z4 Canada; ^3^ Department of Chemical and Biological Engineering University of British Columbia 2360 East Mall Vancouver BC V6T 1Z3 Canada; ^4^ Nanomaterials and Polymer Nanocomposites Laboratory School of Engineering University of British Columbia Kelowna BC V1V 1V7 Canada; ^5^ Basque Centre for Materials Applications & Nanostructures (BCMaterials) Bld. Martina Casiano, 3rd. Floor UPV/EHU Science Park Barrio Sarriena s/n Leioa 48940 Spain; ^6^ Academic Centre for Materials and Nanotechnology AGH University of Krakow Krakow 30‐059 Poland; ^7^ Department of Chemistry The University of British Columbia Vancouver BC V6T 1Z1 Canada

**Keywords:** atmospheric water harvesting (AWH), hierarchical porous structures, metal‐organic frameworks (MOFs), monolith scaffolds

## Abstract

Water scarcity, a critical global challenge, has intensified due to the adverse effects of climate change on ecosystems and its detrimental impact on human activities. Addressing this issue requires solutions capable of providing clean water in regions facing hydroclimatic challenges and limited infrastructure. Atmospheric water harvesting (AWH) offers a promising solution, particularly in arid regions, by extracting moisture from the air. This review explores AWH technologies that leverage material porosity and hygroscopicity, focusing on highly porous materials such as Metal‐Organic Frameworks (MOFs) and monolithic scaffolds. While MOFs exhibit exceptional water uptake due to their tunable chemistry and nanoscale porosity, their powdery nature poses stability and processability challenges. To overcome these limitations, integrating MOFs into multiscale porous monoliths—such as foams, aerogels, cryogels, and xerogels—enhances structural integrity and performance. The role of hierarchical porosity, engineered across nano‐scale in MOF (<2 nm) and micro‐scales (>2 nm) is emphasized in porous monoliths, in optimizing water capture efficiency. This review also highlights recent advancements in MOF‐based composite monoliths, their working mechanisms, and the potential for large‐scale implementation. By integrating nanotechnology with material chemistry, this work outlines strategies to enhance sorption capacity, desorption kinetics, and scalability, ultimately providing a roadmap for developing efficient, sustainable, and scalable AWH systems.

## Graphical abstract

This review explores the design of hierarchical porous materials for atmospheric water harvesting, focusing on metal‐organic frameworks (MOFs) and porous monoliths. Emphasis is placed on integrating MOF nanoscale porosity with the microscale channels of monolithic scaffolds to enhance sorption‐desorption performance. The role of multiscale porosity, from the nanometer scale to the macrostructure, is highlighted in optimizing water uptake, vapor transport, and stability under varying humidity.

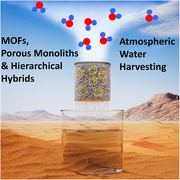

## 1 Introduction

Leonardo da Vinci famously said, “Water is the driving force of all nature.” Today, freshwater scarcity is a significant global challenge, affecting nearly 4 billion people for at least one month each year,^[^ ^1^, ^2^, ^3^ ^]^ with 500 million experiencing severe shortages year‐round.^[^ ^4^ ^]^ Among potential solutions,^[^ ^5^, ^6^, ^7^ ^]^ atmospheric water harvesting (AWH) has gained increasing attention as a method to extract freshwater directly from the atmosphere,^[^ ^8^ ^]^ a vast and renewable water source containing ≈12,900 km^3^ of water vapor.^[^ ^9^, ^10^ ^]^ Unlike conventional water sources,^[^ ^11^ ^]^ atmospheric moisture is globally available, allowing AWH to function independently of surface and groundwater supplies, making it a promising solution for decentralized and off‐grid water generation.^[^ ^12^ ^]^ This approach can be categorized into three main technologies: fog harvesting, dew condensation, and sorption‐based water capture.^[^ ^13^ ^]^ While fog and dew collection require high relative humidity (RH > 70%) and are geographically limited,^[^ ^14^, ^15^, ^16^, ^17^, ^18^, ^19^, ^20^ ^]^ sorption‐based AWH offers a more adaptable and efficient alternative.^[^ ^21^, ^22^, ^23^ ^]^

To date, sorption‐based AWH materials have been developed across three main categories: hygroscopic salts, polymer‐based hydrogels, and highly porous materials such as metal‐organic frameworks (MOFs) and hygroscopic monoliths.^[^ ^24^, ^25^, ^26^, ^27^, ^28^ ^]^ Hygroscopic salts like lithium chloride (LiCl) and calcium chloride (CaCl_2_) demonstrate excellent water uptake due to strong ion‐dipole interactions,^[^ ^29^ ^]^ but their tendency to deliquesce into liquid limits their practical applications due to leakage and handling issues.^[^ ^30^ ^]^ Similarly, polymer‐based hydrogels, known for their high‐water retention capacity, suffer from poor performance at low humidity (<30%), mechanical instability, and degradation over time, making them unsuitable for large‐scale or arid‐environment applications.^[^ ^31^ ^]^

**Figure 1:**
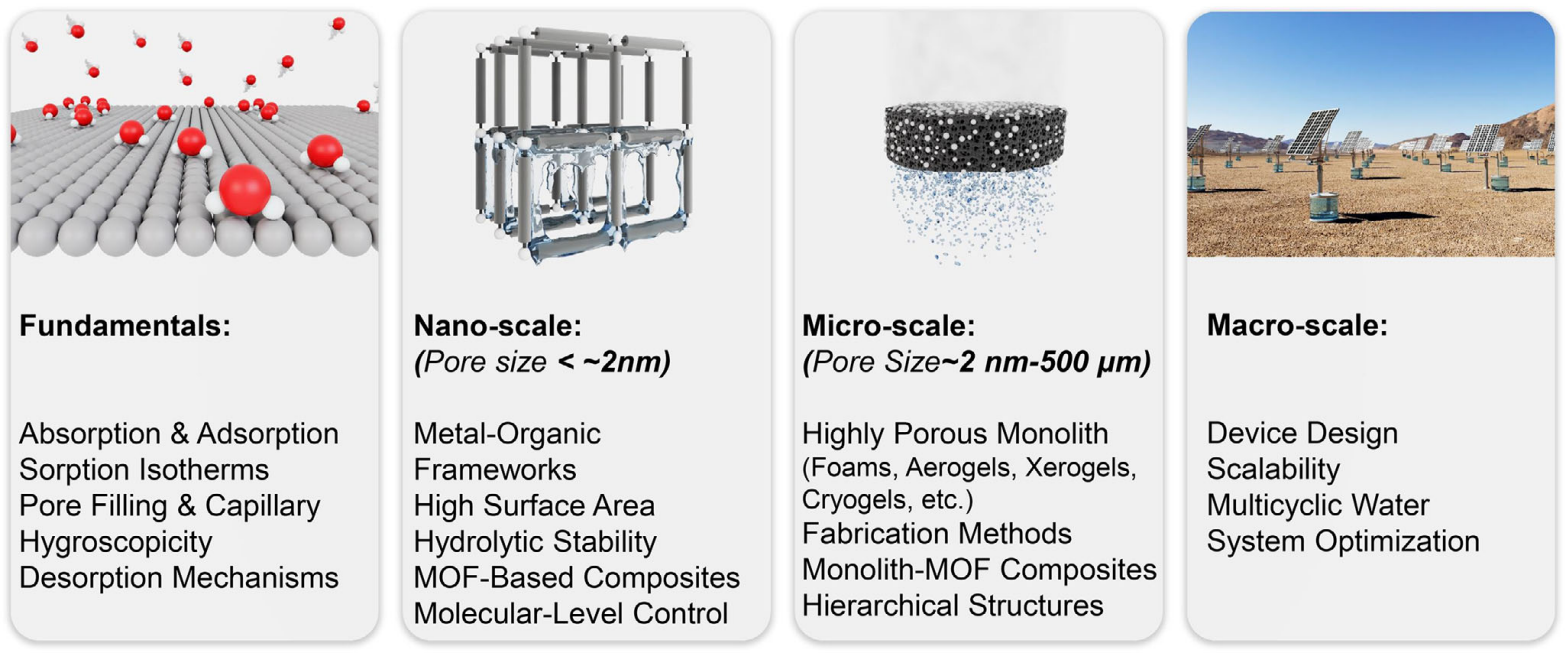
Fundamentals and multiscale integration of porous materials (nano‐ and micro‐scale) for Atmospheric Water Harvesting (AWH), along with macro‐scale device optimization.

On the other hand, porous AWH materials represent a breakthrough in atmospheric water harvesting due to their exceptionally high surface area, which enhances their interaction with water molecules. Their porosity also provides an opportunity to encapsulate other active materials, such as hygroscopic salts, significantly improving their water uptake capabilities.^[^ ^32^ ^]^ These materials are generally classified based on their pore size and structure, each exhibiting distinct sorption mechanisms that can be tailored for AWH applications. While conventional classification schemes categorize pores into micropores (<2 nm), mesopores (2–50 nm), and macropores (>50 nm), this review adopts a modified porosity framework. In this classification, “nano” refers to sub‐2 nm structures that dominate molecular‐level interactions, characteristic of MOF‐based water harvesters. “Micro” describes intermediate‐scale porosity that influences vapor diffusion and retention, primarily seen in porous monolith‐based AWH materials. Finally, “macro” pertains to large‐scale design and instruments, a critical factor in optimizing device‐level water collection and release performance (see **Figure** **1**).

Within this classification, MOFs stand out as a leading class of AWH materials, characterized by pronounced nanoscale porosity and a framework composed of organic and inorganic building units that can be precisely engineered through reticular chemistry.^[^ ^33^, ^34^ ^]^ Their highly porous nature and functionalized metal centers provide strong interactions with water molecules.^[^ ^35^ ^]^ However, despite these advantages, their powdery form,^[^ ^33^ ^]^ water stability concerns, and limited pore connectivity pose challenges for large‐scale implementation.^[^ ^34^, ^36^ ^]^

Beyond MOFs, hygroscopic porous monoliths have gained attention as a promising alternative due to their highly microporous and lightweight structure, which enables efficient water capture and transport. Monoliths often rely on hydrophilic polymer backbones, which not only provide structural integrity but also serve as a medium for encapsulating hygroscopic salts as active water‐capturing sites.^[^ ^37^, ^38^, ^39^, ^40^ ^]^ In addition to their sorption capabilities, porous monoliths exhibit a unique advantage in their compatibility with solar‐driven evaporation strategies—either through direct coating with solar evaporation layers or by hosting solar‐absorbing materials within their structure—enhancing their potential for sustainable water harvesting. However, despite these advantages, porous monoliths often suffer from structural fragility,^[^ ^41^ ^]^ particularly in smaller pores, where high capillary forces induce collapse, undermining their long‐term stability under repeated adsorption‐desorption cycles and reducing their water absorption capability at low relative humidity.^[^ ^42^ ^]^

To address these limitations, MOF‐based composite porous monoliths have recently emerged as a promising class of advanced AWH materials. These hybrids leverage monoliths as structural scaffolds to stabilize MOFs, resolving the handling difficulties of MOF powders. Their multiscale porosity integrates the nanoscale adsorption properties of MOFs, the microporous diffusion channels of porous monoliths, and their networks for mass and heat transfer, creating a synergistic platform capable of efficient water capture across diverse environmental conditions. By combining MOFs and hygroscopic porous monoliths, these composite materials not only improve water uptake under low humidity conditions but also enhance sorption‐desorption efficiency, providing a scalable and robust solution for atmospheric water harvesting.

In this review, we explore the concept of porous materials for AWH, spanning from nanoscale MOF to microporous porous monoliths, ultimately leading to multiscale porous AWH materials engineered through MOF‐based monolith composites. The discussion begins with the fundamentals of AWH, with a primary focus on sorption and desorption mechanisms in materials (Section 2). We then examine nanoporous MOF water harvesters and micro porous monoliths, detailing their underlying water sorption chemistry and mechanisms in Sections 3 and 4, respectively. Finally, we delve into the emerging field of MOF‐based monolith harvesters, highlighting their multiscale porosity and role in advancing AWH efficiency (Section 5), and extend this discussion to the fabrication of macroscale AWH devices (Section 6).

## 2 Fundamentals of AWH

Efficient AWH systems rely on the fundamental processes of sorption (adsorption and absorption),^[^ ^43^ ^]^ desorption,^[^ ^44^ ^]^ and sorption/desorption balance. This section explores how different materials fundamentally capture and release water, examining the role of hygroscopic salts, hydrogels, porous adsorbents (silica gel, zeolites, MOFs, monoliths), and hybrid systems. We also discuss sorption isotherms, which define water uptake behavior and desorption mechanisms, including energy requirements and surface modifications for efficient water release. Finally, we address the sorption/desorption ratio, a key factor in ensuring sustainable and high‐performance AWH.

### 2.1 Sorption, Absorption, and Adsorption in AWH

**Figure 2:**
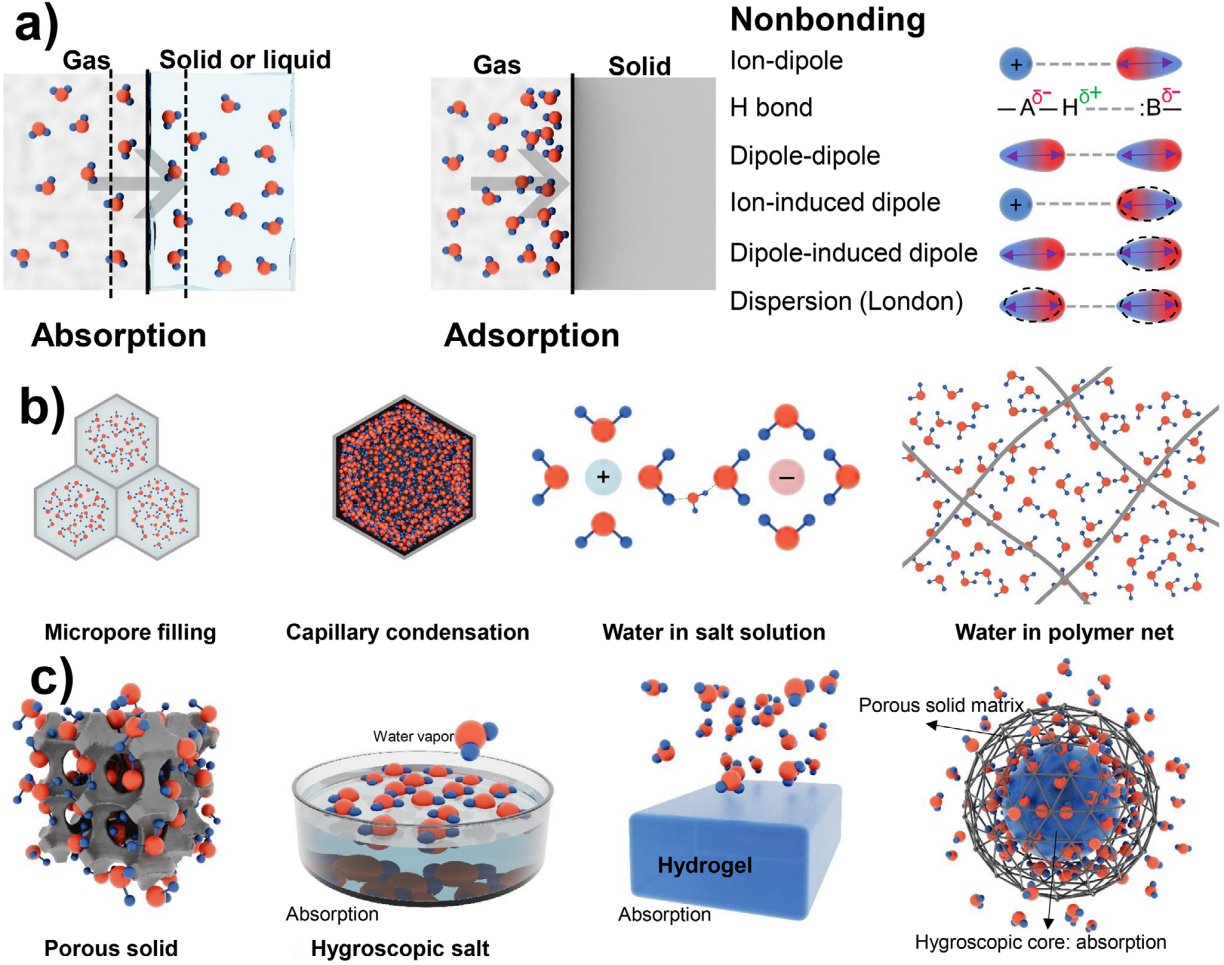
Schematic illustration of water molecules a) absorption versus adsorption and possible interactions with other elements in their vicinity; b) ad‐/absorption mechanism in different environments: pore filling and capillary condensation in porous materials, salt solution and polymer network; and c) comparison of the adsorption and absorption phenomena in different structures of sorbents, like porous solid, hygroscopic salt, hydrogel, and composites.

The fundamental process in AWH begins with sorption, the uptake of water molecules by a material. This process is broadly categorized into adsorption and absorption,^[^ ^45^ ^]^ two mechanisms that describe distinct interactions between the water molecules and the sorbent. Adsorption occurs when water molecules adhere to the surface of the material, including both external surfaces and internal pore structures, whereas absorption involves the penetration of water molecules into the bulk structure of the sorbent.^[^ ^46^ ^]^ These mechanisms, illustrated in **Figure** **2**a, govern the efficiency and functionality of AWH materials and dictate their applicability across different environmental conditions. Understanding the interplay between adsorption and absorption is crucial for optimizing water sorption materials, particularly those designed to operate effectively across varying humidity levels.

Adsorption occurs when water molecules, the adsorbate, bind to the surface of the adsorbent, through non‐bonding interactions such as van der Waals forces, hydrogen bonding, ion‐dipole, and dipole‐dipole interactions. These weak intermolecular forces allow water molecules to adhere the surface structure, making surface chemistry and porosity critical factors in adsorption efficiency.^[^ ^47^ ^]^ Porous materials excel as adsorbents due to their high surface area, which provides extensive binding sites for water molecules. Within these porous structures, two key mechanisms—pore filling and capillary condensation—enhance adsorption performance.

Capillary condensation occurs when confined vapor in small pores forms a curved liquid‐vapor interface, lowering the equilibrium vapor pressure and inducing condensation at humidity levels lower than the saturation vapor pressure.^[^ ^48^ ^]^ This process is particularly dominant in micropores (>2 nm), where water condenses into liquid, optimizing adsorption efficiency (Figure 2b). By contrast, pore filling involves a gradual accumulation of water molecules along the internal surfaces of smaller pores, particularly in nanoporous adsorbents (<2 nm), where overlapping pore walls exert strong attractive forces. This allows for effective water capture even at ultralow RH,^[^ ^49^ ^]^ making nanoporous materials highly efficient for AWH applications.^[^ ^50^ ^]^

Depending on the nature of the interactions, adsorption can be classified into physisorption and chemisorption.^[^ ^51^ ^]^ Physisorption is characterized by weak intermolecular forces and is typically reversible, allowing for easy desorption and regeneration of the material. In contrast, chemisorption involves the formation of strong chemical bonds between the adsorbate and the adsorbent, often leading to irreversible water uptake.^[^ ^52^, ^53^ ^]^ The energy required for desorption varies significantly between these two modes, influencing the material's performance under cyclic AWH conditions. The desorption characteristics of adsorbents will be further explored in subsequent sections.

Unlike adsorption, absorption involves the uptake of water molecules into the bulk structure of the material rather than just the surface (Figure 2a). This process occurs in materials capable of swelling or retaining water within their internal matrix, such as hydrophilic polymers or sponges, which accommodate large volumes of water through diffusion‐driven transport. During absorption, water molecules experience mass transfer into the material, propelled by a concentration gradient that enables deeper penetration into the sorbent (Figure 2b). The kinetics of absorption tend to be slower than adsorption due to the time required for water molecules to diffuse into the absorbent's structure.

Pore filling also plays a crucial role in absorption, where the pores within the material become saturated with water. In some cases, capillary condensation further facilitates the process, particularly in materials with well‐defined porosity, enhancing water uptake under moderate humidity conditions. However, unlike adsorption, absorption often involves deeper integration of water into the material's framework, which can make desorption more dependent on structural properties and external forces. While some absorbed water can be easily removed (e.g., by squeezing a sponge), in other cases, it is less reversible due to strong interactions within the material. This is particularly evident in materials such as hydrogels, where absorbed water becomes chemically or physically entrapped within the polymeric matrix (Figure 2c). The distinction between adsorption and absorption is particularly important when designing AWH materials, as their effectiveness depends on whether rapid surface interactions (adsorption) or deep bulk penetration (absorption) are more suitable for the intended application.^[^ ^54^ ^]^

Both adsorption and absorption contribute to the water harvesting capabilities of various materials, but their efficiency depends on material composition, pore structure, and environmental conditions. By integrating hierarchical porosity and tuning surface chemistry, researchers can optimize AWH materials to balance water uptake capacity, desorption efficiency, and mechanical stability. Understanding these sorption mechanisms is essential for designing next‐generation AWH materials that perform efficiently across a broad spectrum of humidity conditions, particularly in arid environments where water capture is most challenging.

### 2.2 Adsorption and Absorption in Different AWH Materials

Understanding the mechanisms of adsorption and absorption in various sorbent materials is essential for advancing AWH technologies. Each class of sorbents—including hygroscopic salts, polymer‐based hydrogels, and porous materials such as silica gel, zeolites, MOFs, and monoliths—offers unique water uptake pathways dictated by distinct physicochemical principles.^[^ ^55^ ^]^ These materials differ in their structure, water affinity, and sorption behavior, ultimately influencing their efficiency in capturing and releasing moisture. The potential of these materials in AWH is summarized in **Table** **1**, while their specific roles are explored in detail below.

**Table 1:** Compares different sorbent materials from different perspectives.

	Porous Solid sorbent (Class 1)	Porous Solid sorbent (Class 2)	Hygroscopic salts	Polymer‐based sorbent (Hydrogel)
System	MOFs, COFs, Silica gels, Zeolites	Aerogel Xerogel Cryogel Monolithic scaffolds	LiCl, CaCl_2_, LiBr, MgCl_2_	PAM, PAAS, PNIPAAM, Cellulose
Sorption mechanism	Adsorption, pore filling, Capillary condensation	Adsorption, pore filling, Capillary condensation	Absorption	Absorption
Onset sorption humidity	Low (<30% RH)	Low to medium (30%≤RH≤60)	Low to medium (30%≤RH≤60)	Low to medium (30%≤RH≤60)
Sorption capacity	Low	Moderate	High	High
Sorption kinetics	Fast	Moderate to Slow	Slow	Slow
Sorption isotherm	S‐shaped	Type IV or V	Linear or quasi‐linear	Linear or quasi‐linear
Advantages	Structural tunability Physiochemical properties	High porosity Low density Encapsulation capability	Availability Easy synthesis Comprehensive mechanisms	Rich chemistry Functionalization Stimuli‐responsive
Disadvantages	Powdery form Complex synthesis	Fragility	Agglomeration Leakage Corrosion	Relatively new Less understood mechanism

Abbreviations: MOFs: Metal‐Organic Frameworks, COFs: Covalent Organic Frameworks, LiCl: Lithium Chloride, CaCl_2_: Calcium Chloride, LiBr: Lithium Bromide, MgCl_2_: Magnesium Chloride, PAM: Polyacrylamide, PAAS: Poly(acrylic acid) sodium salt, PNIPAAM: Poly(N‐isopropylacrylamide), RH: Relative Humidity.

Hygroscopic salts, such as lithium chloride (LiCl), calcium chloride (CaCl_2_), lithium bromide (LiBr), and magnesium chloride (MgCl_2_), represent a key class of absorbents with a high intrinsic affinity for water. Their water uptake occurs through hydration and deliquescence, processes governed by the difference in vapor pressure between the salt surface and the surrounding air. As water molecules integrate into the salt crystals, they either bind within the crystal lattice or form a concentrated saline solution, where water molecules coordinate around the salt ions.^[^ ^56^ ^]^ While these materials offer exceptional water uptake capacities, they also suffer from several drawbacks, including the formation of an inactive surface layer that inhibits further absorption, particle agglomeration, leakage upon deliquescence, and corrosive effects that limit their practical application.^[^ ^30^ ^]^ These challenges necessitate further improvements to enhance their long‐term performance in AWH systems.

Polymer‐based hydrogels, another category of absorbents, have gained attention due to their hydrophilic functional groups (‐NH₂, ‐OH, ‐COOH, ‐SO_3_H), which allow them to swell and retain large amounts of water. These materials consist of cross‐linked polymeric networks capable of trapping water clusters within their structure, making them highly efficient for moisture absorption and storage. The water retention capacity of hydrogels can be fine‐tuned by modifying the cross‐linking density or introducing interpenetrating polymer networks (IPNs)^[^ ^31^ ^]^ to enhance water diffusion and stability.

Porous materials, which are widely utilized in AWH, can be categorized based on their backbone sorbent composition and the size of their porosity. As previously discussed, the new generation of porous materials —MOFs—possess nanoscale porosity^[^ ^57^ ^]^ and will be covered extensively in later sections. Beyond MOFs, traditional porous materials, such as silica gel and zeolites, remain relevant due to their well‐defined microporous structures, which vary depending on synthesis conditions. These materials have been extensively used in AWH due to their large surface areas and abundant adsorption sites, which enhance their ability to capture water molecules. Silica gel and zeolites, in particular, contain surface hydroxyl (‐OH) and aluminum (‐Al) functional groups, respectively, which facilitate strong interactions with water molecules.^[^ ^58^ ^]^ Their adsorption mechanism primarily relies on pore filling and capillary condensation, effectively increasing water uptake efficiency. However, despite their high hydrophilicity, these materials exhibit limitations such as low total water capacity and slow uptake kinetics. Furthermore, their high desorption energy requirements—due to the strong bonding between water molecules and the sorbent surface—pose challenges for low‐energy regeneration, restricting their large‐scale implementation.

In addition to silica gel and zeolites, another important class of porous materials is hygroscopic monoliths composed of hydrophilic polymers. These materials differ significantly from conventional porous adsorbents in pore size distribution, AWH mechanisms, and overall water affinity, sometimes leading to their classification as a distinct category. Unlike traditional porous materials, hygroscopic monoliths are characterized by a microporous structure that enhances capillary‐driven absorption while allowing for rapid moisture transport. Additionally, their hydrophilic polymeric backbones not only serve as structural support but also facilitate water sorption through functional groups capable of hydrogen bonding. These monoliths can be further optimized by encapsulating hygroscopic salts within their porous network, effectively combining the high‐water uptake potential of salts with the structural integrity and tunable porosity.

To overcome the individual limitations of different categories of materials, researchers have explored the development of hybrid systems,^[^ ^59^ ^]^ which integrate multiple materials to enhance water uptake efficiency and stability (Figure 2c). For instance, impregnating MOFs or monoliths with hygroscopic salts allows these systems to harness the high‐water sorption capacity of salts while mitigating their major drawbacks, such as leakage and agglomeration. Similarly, combining MOFs with monoliths leverages the high adsorption efficiency of MOFs with the structural integrity of monoliths, addressing the powdery nature of MOFs and the lower water uptake capacity of certain polymers at low humidity. These hybrid systems demonstrate synergistic effects, leading to improved water uptake performance,^[^ ^60^ ^]^ enhanced mechanical stability, and greater applicability across varying environmental conditions.^[^ ^61^ ^]^

By integrating multiple sorption mechanisms and optimizing material properties, hybrid systems offer a promising pathway toward more efficient and scalable AWH technologies. Future advancements in material design, hierarchical porosity engineering, and hybridization strategies will play a crucial role in enhancing water harvesting efficiency while reducing energy requirements for desorption and regeneration, paving the way for next‐generation AWH systems.

### 2.3 Isotherms of Sorption Behavior

Understanding the sorption behavior of materials requires recognizing that different classes of materials exhibit distinct sorption characteristics. This behavior is mathematically described by the relationship between equilibrium water uptake and RH at a given temperature. To classify these behaviors, the International Union of Pure and Applied Chemistry (IUPAC) has identified primary isotherm patterns as follows:^[^ ^62^ ^]^

Type I isotherms, typically described by the Langmuir model, represent monolayer adsorption on homogeneous surfaces, such as activated carbons and zeolites.^[^ ^63^, ^64^, ^65^, ^66^ ^]^ These isotherms are characterized by an initial steep rise, which reflects the rapid occupation of high‐affinity adsorption sites. As these sites become saturated, the curve levels off, indicating the limit of monolayer adsorption capacity. However, their limited water uptake at higher humidity levels restricts their effectiveness in environments with fluctuating humidity.^[^ ^67^ ^]^

Type II isotherms correspond to multilayer adsorption on nonporous or macroporous materials. Initially, the curve resembles a Type I isotherm, indicating monolayer adsorption. However, beyond a characteristic “knee” point, the adsorption process continues, demonstrating the onset of multilayer adsorption. Unlike Type I, Type II isotherms do not reach a plateau, as additional water molecules continuously accumulate on the surface.

Type III isotherms indicate weak interactions between the adsorbate and adsorbent.^[^ ^68^ ^]^ At low pressures, the adsorption rate remains minimal due to weak binding forces. However, as the pressure increases, multilayer adsorption becomes dominant, resulting in a gradual increase in adsorption capacity.

Types IV and V isotherms, both distinguished by the presence of a hysteresis loop, are associated with capillary condensation within mesopores. Type IV isotherms reflect strong adsorbate‐adsorbent interactions, beginning with monolayer adsorption, followed by multilayer adsorption, and eventually transitioning into capillary condensation. The “knee” point in Type IV marks the transition from multilayer adsorption to capillary condensation. Type V isotherms, in contrast, exhibit weaker adsorbate‐adsorbent interactions, initially resembling Type III behavior. However, at higher pressures, the presence of a hysteresis loop indicates the onset of capillary condensation within mesopores.^[^ ^69^, ^70^ ^]^

Finally, Type VI isotherms depict stepwise multilayer adsorption, where each distinct “step” corresponds to the completion of one adsorbed water layer before the next begins. This stepwise behavior arises due to strong intermolecular forces between adsorbed water molecules, leading to well‐defined transitions between adsorption layers. This classification of isotherms provides critical insights into how different AWH materials interact with water vapor, allowing for the design of optimized sorbents that perform effectively across varying environmental conditions.

### 2.4 Desorption

In AWH, desorption is pivotal, involving the release of water from materials. This process hinges on fundamental sorption mechanisms—adsorption and absorption—which dictate the ease with which water molecules are released.^[^ ^71^ ^]^ Physisorption typically involves weaker, surface‐level interactions such as van der Waals forces or hydrogen bonding, making water relatively easier to release. In contrast, chemisorption occurs when water molecules are absorbed through stronger interactions, such as ion‐dipole forces, potentially making desorption more difficult. This distinction highlights a critical challenge in water release: materials with high water affinity, such as pure salts, often struggle to desorb water due to strong ion‐dipole interactions. Conversely, MOFs can be engineered to facilitate easier water release through tunable chemistry and pore design.^[^ ^72^ ^]^ To facilitate the desorption process, it is essential to consider the enthalpy of evaporation, which is influenced not only by the material's chemistry but also by its structural properties.^[^ ^73^ ^]^

**Figure 3:**
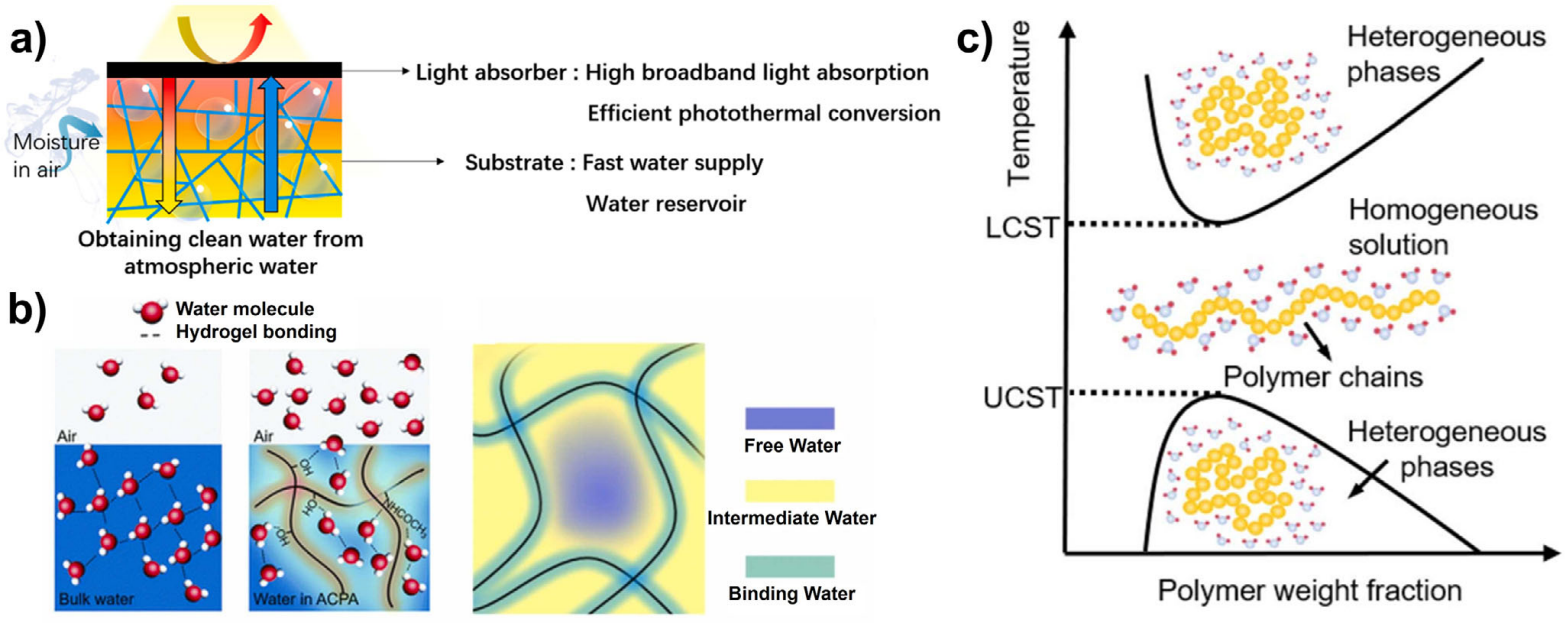
a) Solar‐powered water desorption derived from solar‐thermal conversion of light absorber materials; b) fraction of different states of water schematic demonstrating hydrogen bond theory (Reproduced with permission.^[^ ^86^ ^]^ Copyright 2023, Elsevier); c) phase separation diagram of thermal response of polymers’ phase separation (upper critical solution temperature (UCST) and lower critical solution temperature (LCST) utilized as a water desorption mechanism (Reproduced with permission.^[^ ^48^ ^]^ Copyright 2023, Royal Society of Chemistry).

The desorption process in porous media is governed by enthalpy (ΔH) and entropy (ΔS). Breaking hydrogen bonds or van der Waals forces requires energy, making ΔH positive. As water molecules transition from a confined, ordered state within pores to a more disordered gas phase, ΔS increases, favoring desorption (ΔG = ΔH − TΔS).^[^ ^74^ ^]^ However, in highly confined systems with strong hydrogen bonding, the increase in ΔS is limited, making desorption less spontaneous unless the temperature is raised, mostly with solar energy (**Figure** **3**a).

Water molecules in porous systems can be classified into free water (FW), intermediate water (IW), and bound water (BW)^[^ ^75^ ^]^ as shown in Figure 3b. FW is weakly bound and evaporates easily, IW interacts with the surface but retains some mobility, and BW is strongly held within the material's structure. These classifications are crucial for understanding desorption efficiency, as FW requires less energy for desorption, while BW requires significantly more energy due to stronger adhesive forces. In some porous materials, the ΔH of evaporation can decrease due to weaker interactions. Surface functionalization—such as adding hydrophobic groups—reduces water‐surface adhesion, lowering the energy needed for desorption.^[^ ^76^ ^]^ Thus, materials like MOFs and functionalized monoliths, which weaken water interactions, exhibit lower ΔH, improving water release efficiency.^[^ ^76^ ^]^

A double‐layer technique (Figure 3a) is often used to optimize light absorption and heat retention, accelerating evaporation. Photothermal materials convert solar radiation into heat, facilitating water release.^[^ ^73^ ^]^ Carbon‐based materials, such as carbon nanotubes (CNTs), MXenes, and carbonized polymers, are particularly effective due to their ability to convert solar energy into localized heat.^[^ ^77^, ^78^ ^]^ Polymers like polypyrrole (PPY), polyaniline (PANI), and polydopamine also exhibit photothermal properties, enhancing desorption.^[^ ^79^ ^]^ However, photothermal desorption depends on solar availability, limiting its function to daylight hours. To enable desorption independent of solar energy, localized electrical heating (LEH) provides fast,^[^ ^80^ ^]^ and uniform desorption via Joule heating.^[^ ^81^ ^]^

Another strategy for water release is hydrophilicity switching, which modifies surface properties to alter water affinity.^[^ ^82^ ^]^ By shifting from a hydrophilic to a hydrophobic state, materials spontaneously release water without requiring additional energy input.^[^ ^83^ ^]^ A key example is Lower Critical Solution Temperature (LCST) behavior (Figure 3c). Above the LCST, polymer chains transition from hydrophilic coils to hydrophobic globules, leading to water ejection/expulsion. Poly(N‐isopropylacrylamide) (PNIPAAM), which releases water above 35 °C, is a prime example of this mechanism.^[^ ^84^ ^]^ This approach is able to enhance desorption efficiency,^[^ ^85^ ^]^ making porous materials highly effective for sustainable water management.

### 2.5 Sorption/Desorption Ratio

AWH operates as a cyclic process, alternating between sorption (water uptake) and desorption (water release). The efficiency of the system depends not only on the amount of water absorbed or adsorbed but also on the amount effectively released during desorption. An essential metric for evaluating this efficiency is the sorption/desorption ratio (often called AWH efficiency), with an ideal value close to 1. A ratio significantly greater than 1 indicates hysteresis, where water remains bound to the sorbent due to strong interactions such as hydrogen bonding and ionic interactions with hydrophilic sites.^[^ ^87^ ^]^ Hysteresis reduces the system's performance over multiple cycles by trapping residual water within the material.

In the context of porous sorbents, pore structure and size play a crucial role in sorption/desorption efficiency. The size, distribution, and connectivity of pores influence how efficiently water is sorbed and released.^[^ ^88^ ^]^ Optimizing pore size and surface properties helps reduce hysteresis, ensuring a balanced sorption/desorption ratio and maintaining high efficiency over repeated cycles.^[^ ^89^ ^]^ All combined, the efficiency of AWH systems is inherently linked to pore structure and chemistry engineering across different scales.

## 3 Pure MOFs and Their Non‐Monolith Composites for Atmospheric Water Harvesting

In the previous section, MOFs were introduced as promising materials for AWH due to their highly tunable chemistry, which can be precisely tailored to enhance interactions with water molecules. Their exceptional performance in capturing water, even at low relative humidity, further underscores their potential for AWH applications. In the following sections, we provide a concise overview of MOFs, the fundamental mechanisms governing their water sorption behavior, and key chemical factors influencing their adsorption performance. Additionally, we discuss non‐monolith MOF composites, including MOF‐salt hybrids and MOF supraparticles, which offer innovative pathways for next‐generation AWH systems.^[^ ^86^ ^]^

### 3.1 MOF Chemistry and Structure: A Reticular Approach to Tunable Nano‐scale Porosity

Imagine a 3D puzzle, where the pieces are intricately designed inorganic metal nodes and organic chemical linkers. These elements come together as the fundamental building blocks,^[^ ^90^ ^]^ crafting the molecular‐level framework of MOFs. When thousands of these “puzzle pieces” unite, they give rise to stable MOFs, each with its unique shape and size, determined by the choice of building units and topology.^[^ ^35^ ^]^ These remarkable characteristics allow MOFs to possess customizable porous structures, achieved by adjusting the length of chemical linkers to modify crystal size, resulting in an exceptionally large surface area compared to other porous materials.^[^ ^91^ ^]^ Within these cages, nanoscale pores, measuring less than a few nanometers, contribute to MOFs' remarkable porosity, low density, good water uptake capacity, and extreme sorption/desorption ratio. The field of reticular chemistry facilitates the meticulous assembly of MOFs from the ground up, providing a versatile palette of metal nodes (or secondary building units (SBUs)) and chemical linkers. The result is a vast spectrum of MOFs, each with distinct chemical traits like surface charge and functional groups, as well as physical properties such as size and morphology.^[^ ^92^ ^]^

MOF formation relies on nucleation and growth. Inorganic building units, known as SBUs, initiate nucleation, while organic linkers drive growth, forming an extended crystalline porous structure. Generally, narrow distribution in the size and morphology of MOFs originates from fast nucleation and slow growth.^[^ ^33^ ^]^ Various synthetic parameters also impact MOFs' chemical and physical properties, and controlling these parameters aids in achieving desired MOFs with specific porous structures, particularly in water harvesting applications.^[^ ^93^ ^]^ **Table** **2** displays key parameters and their impacts on MOFs' properties.

**Table 2:** Effective parameters in MOF synthesis.

Parameter	Affected by	Impacts on	Advantages and Disadvantages
Nature of Metal Nodes and Chemical Linkers	–	Chemical and physical properties	*Advantages*: Diversity, tunable chemical and physical features. *Disadvantages*: Complexity in synthesis.
Ratio of Metal Nodes to Chemical Linkers	–	Size and size distribution of nanoparticles	*Advantages*: Controllable (low ratio, high nucleation rate, small size, broad size distribution). *Disadvantages*: Requires precise control during synthesis.
Solvent	–	Reactivity of linkers	*Advantages*: Controllable by choosing the appropriate solvent based on counterions. *Disadvantages*: Improper solvent can slow down the reaction and cause aggregation.
Modulator	–	Crystallinity, morphology, and size of nanoparticles	*Advantages*: Modulates reactivity of building units, and controls crystal growth. *Disadvantages*: Often requires trial‐and‐error to optimize.
Reaction Time	Synthesis method (micro‐/macro‐confined approaches)	Morphology and size of nanoparticles	*Advantages*: Can fine‐tune nanoparticle properties. *Disadvantages*: Affected by heat transport, requiring careful management.
Heat/Temperature	Synthesis method (micro‐/macro‐confined approaches)	Morphology and size of nanoparticles	*Advantages*: Enables control over synthesis conditions. *Disadvantages*: Difficult to control uniformly; reactor type affects homogeneity, leading to size variation.
Mixing Degree	Synthesis method (micro‐/macro‐confined approaches)	Morphology and size of nanoparticles	*Advantages*: Ensures homogeneous solution and uniform particle size. *Disadvantages*: May impact heat transport, affecting final nanoparticle size.

Post‐synthetic modification (PSM) techniques allow for the full utilization of MOFs' potential by further refining their physical and chemical features according to specific demands.^[^ ^94^ ^]^ Through PSM, metal nodes and/or linkers can also be exchanged.^[^ ^95^ ^]^ These modifications result in a wide range of MOFs with fine‐tuned final properties. Therefore, MOFs can be precisely customized, not only by adjusting synthetic parameters but also through PSM methods. It is important to note that all these alterations can have an impact on the porous structure of the final framework.^[^ ^96^ ^]^

### 3.2 Mechanisms Underpinning MOF Capabilities for AWH

In general, the adsorption mechanisms of MOFs, dictated by their structural characteristics, can be categorized into three main types: (I) chemisorption at open metal sites, (II) physisorption through layer or cluster formation, and (III) capillary condensation.^[^ ^93^ ^]^ Additionally, in the case of MOFs with flexible frameworks, another mechanism known as breathing may influence water adsorption behavior. This section will explore these mechanisms in detail, highlighting their impact on the water‐harvesting capabilities of MOFs.

I) *Chemisorption on open metal sites*: The SBU skeleton of MOFs is made up of metal ions with high coordination capacities, such as Mg^2+^, Zn^2+^, Ni^2+^, and Co^2+^. Neutral donor ligands act as complementary elements, connecting to the metal core of paddle‐wheel or rod‐like SBUs. Under vacuum and heating, these ligands detach, leaving unsaturated coordination sites. In this situation, water molecules from the ambient air can replace the missing ligand, completing the coordination sphere of the metal center.^[^ ^97^ ^]^ This chemisorption process leads to a Type I isotherm, meaning that water adsorption occurs at very low relative humidity. The binding strength between water molecules and the open metal sites depends on the electronegativity of the metal ion, requiring high regeneration temperatures for desorption. Over multiple adsorption‐desorption cycles, this strong chemisorption can induce structural deformation, leading to gradual material degradation.^[^ ^98^ ^]^ As a result, some MOFs with open metal sites exhibit hysteresis loops in their isotherms, indicating differences between adsorption and desorption behavior due to the strong binding forces at the metal sites.
II) *Physisorption in the form of layers or clusters*: In this reversible mechanism, polar and hydrophilic centers in different regions of the microporous structure act as nucleation sites, initiating water adsorption.^[^ ^99^ ^]^ These nucleation sites are defined by the average of polar momenta.^[^ ^100^ ^]^ Once water molecules adsorb, they serve as additional adsorption sites, leading to the growth of water clusters.^[^ ^101^ ^]^ When these small clusters connect, continuous pore‐filling occurs.^[^ ^102^ ^]^
III) *Capillary condensation*: This irreversible and hysteretic adsorption mechanism occurs in nano‐porous MOFs with pore diameters exceeding a critical value (D_c_), according to Equation (1):^[^ ^103^ ^]^
(1) $$D_{c}=\frac{4\sigma T_{c}}{\left(T_{c}-T\right)}$$

here σ is the van der Waals diameter of the adsorbate (water), and T_c_ and T are the critical temperature of the water and the adsorption temperature, respectively. This critical diameter is 20.76 Å at 25 °C, though this value varies with atmospheric temperature. Capillary condensation can be minimized by adjusting the pore size and shifting adsorption to higher temperatures.^[^ ^104^ ^]^ This mechanism produces an S‐shaped isotherm with a hysteresis loop, indicating that not all pore capacity is accessible.^[^ ^105^ ^]^ The strong adsorbent‐adsorbate interactions result in residual water retention within the MOF structure even after desorption. Furthermore, hysteresis loops may also appear in pores smaller than 20 Å due to structural deformation or degradation rather than capillary condensation.^[^ ^106^ ^]^

**Figure 4:**
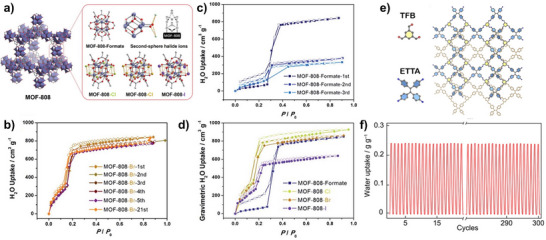
a) 3D architecture of MOF‐808 after the replacement of formate groups with halide ions in its structure; b) stability of water uptake capacity of MOF‐808‐Br throughout 21 cycles compared with c) that of MOF‐808‐formate after the first 3 cycles; d) water uptake diagram of MOF‐808 before and after modification (Reproduced with permission.^[^ ^107^ ^]^ Copyright 2022, Royal Society of Chemistry); e) covalent attachment of 1,1,2,2‐tetrakis(4‐aminophenyl) ethane (ETTA) and 1,3,5‐triformylbenzene (TFB) yielding COF‐432; f) Brilliant water cycling stability of COF‐432 after 300 cycles of adsorption at 40% RH (at 30°C) and desorption at 30% RH (at 30°C) (Reproduced with permission.^[^ ^108^ ^]^ Copyright 2020, American Chemical Society).

So far, various techniques have been introduced to solve the hysteresis problem. One promising strategy is reinforcing the MOF's structure to tolerate tensions during the cyclic adsorption‐desorption. In this regard, Lu et al.^[^ ^107^ ^]^ chose a widely available Zr‐based MOF named MOF‐808, which is synthesized by using formic acid as a modulator, and tried to replace unwanted formate ligands with halide ions. For this reason, they treated formate‐afflicted MOF‐808 with an aqueous solution of HCl, HBr, and HI in the presence of dioxane. This way, formate groups were replaced with terminal aqua ligands in a halo‐acid treated MOF‐808 structure, depicted in **Figure** **4**a. The cyclic water uptake results of formate‐afflicted MOF‐808‐Br and nonmodified MOF‐808 have been shown in Figure 4b,c. Noteworthy, after this modification, water uptake capacity loss vanished compared to the first harvesting cycle and surprisingly shifted AWH to lower partial pressure, as illustrated in Figure 4d. This result strongly proved the absence of capillary‐force‐induced pore collapse and mechanical stability in the nanoscale. With the aid of this modification, pore‐filling water molecules tend to undergo hydrogen bonding with free halide ions rather than the pore wall, significantly resulting in the reduction of capillary‐force‐induced stress during water release.

In another study by Nguyen et al.,^[^ ^108^ ^]^ a highly crystalline covalent organic framework (COFs, a subclass of MOFs that lacks metal nodes, was synthesized from tetratomic 1,1,2,2‐tetrakis(4‐aminophenyl)ethene [ETTA, (C_26_H_16_(NH_2_)_4_ and tritopic1,3,5‐triformylbenzede {[(ETTA)_3_(TFB)_4_]_imine_} termed COF‐432, whose chemical structures are represented in Figure 4e. This voided square grid exhibits the maximal water uptake of 0.3 g/g at 95% RH, which was 0.23 g/g at 20‐40% RH without hysteretic behavior. The imine groups of this COF are responsible for water molecule adsorption thanks to the hydrogen bond between them. In addition, this hydrogen bonding aligned with the robust confinement effect of the covalently porous framework, setting the stage for adsorption at low RH (<40%) as well as prevention of pore collapse.^[^ ^109^ ^]^ These factors contribute to the excellent working capacity of COF‐432, even after 300 cycles of adsorption/desorption (Figure 4f).^[^ ^108^ ^]^

IV) *Breathing*: A unique adsorption mechanism in MOFs is the breathing effect, which is particularly relevant for flexible MOF structures.^[^ ^110^ ^]^ Breathing refers to the reversible expansion and contraction of the MOF framework in response to external stimuli such as pressure, temperature, or guest molecule adsorption (e.g., water molecules). This dynamic behavior allows MOFs to adjust their pore structure, optimizing adsorption capacity under varying environmental conditions. For instance, a recent study demonstrated that guest‐induced breathing in MIL‐88A(Fe) MOFs facilitated the selective recovery of alcohols from aqueous solutions, showcasing the material's ability to expand and contract to efficiently accommodate and release different guest molecules.^[^ ^111^ ^]^ In the context of AWH, the breathing mechanism is particularly advantageous for water adsorption and desorption. Andreo et al.^[^ ^112^ ^]^ reported that a specific MOF exhibited significant breathing effects upon exposure to water vapor, which not only expanded its pore size, enhancing water uptake, but also facilitated easier water release during desorption cycles.

### 3.3 Key Factors Influencing the AWH Performance of MOFs

#### 3.3.1 Hydrolytic Stability of MOFs

In AWH applications, the hydrolytic stability of MOFs is crucial, as they are in direct contact with water molecules. This stability refers to the MOF framework's resistance to degradation when exposed to water.^[^ ^113^ ^]^ Understanding MOF degradation mechanisms is essential, as they directly impact material performance and longevity. MOF degradation occurs through two primary mechanisms: hydrolysis and linker displacement. Hydrolysis involves the cleavage of metal‐linker bonds by OH⁻ groups, leading to the release of a free protonated linker, as shown in Equation (2). In contrast, linker displacement occurs when a water molecule inserts into the MOF structure, resulting in the formation of a free deprotonated linker, as described in Equation (3).^[^ ^114^ ^]^

(2)

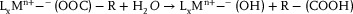



(3)

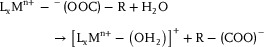

here, L, M, and R represent the organic linker within the MOF structure, the metal ion within the SBU of the MOF, and the organic group connected to the metal‐linker bond, respectively.

The stability of MOFs is influenced by thermodynamic and kinetic factors, making precise control essential for developing hydrolytically stable materials. Thermodynamic stability primarily depends on two key factors: the strength of the metal‐linker bond and the relative energy levels of the metal ions' frontier orbitals compared to those of water. In MOFs, the metal‐linker bond is often the weakest point in the extended framework, despite being formed by chemically stable linkers and SBUs. While bond strength generally correlates with hydrolytic stability, exceptions exist. The interaction between organic linker binding groups and metal centers follows a Lewis acid‐base pair interaction, where stability is influenced by the linker's basicity and the metal ion's acidity.^[^ ^93^ ^]^ This principle is particularly useful in designing thermodynamically stable MOFs, such as pyrazole‐based frameworks, which exhibit high chemical stability due to their robust metal‐linker interactions.

The pKa values of metal centers, estimated based on their charge and radius, are a valuable tool for designing stable MOFs. Metals with high charges, like Ti^+4^, Zr^+4^, and Hf^+4^, tend to form highly stable frameworks.^[^ ^115^ ^]^ Furthermore, metal polarizability plays a crucial role in predicting stability more precisely. The hard‐soft acid‐base (HSAB) theory simplifies the selection of metals and binding groups, stating that strong bonds form when frontier orbitals of similar size and polarizability overlap. Consequently, stable MOFs are typically composed of hard acids (metals) and hard bases (binding groups with high pKa values), leading to strong bonds commonly found in carboxylate, pyrazole, or tetrazole linkers. Considering the abovementioned aspects of MOFs for water adsorption application, we conclude that tuning the required properties for unsaturated AWH highly depends on structural elements such as linker, SBU, and defects.

#### 3.3.2 Impact of MOF Linker

The exposed surface area (van der Waals surface) and the polar surface of an organic linker play a key role in determining the hydrophilicity or hydrophobicity of MOF pores. Large, nonpolar linkers (with a high A_vdW_/A_polar_ ratio) create hydrophobic pores, while small, polar linkers (with a low A_vdW_/A_polar_ ratio) lead to hydrophilic pores.^[^ ^93^ ^]^ Typically, polar molecules capable of hydrogen bonding are considered hydrophilic, whereas certain functional groups, such as nitro groups, esters, and ketones, are “hydroneutral”, meaning they neither attract nor repel water significantly.^[^ ^116^ ^]^ The hydrophilicity of pores directly influences the isotherm behavior of MOFs, shifting the inflection point toward lower humidity levels, which is advantageous for AWH applications in arid regions.^[^ ^117^ ^]^

With the idea of linker design, Zheng et al.^[^ ^118^ ^]^ utilized artificial intelligence (AI) to explore a wide range of linker backbones and conformational alterations of secondary building units, leading to the development of ten types of Al‐based long‐arm MOFs (LAMOFs). In this research, by substituting the pyrazole ring with thiophene, furan, thiazole, or their combinations, they created MOFs capable of operating efficiently in low to moderate RH (13%–53%), achieving high water uptake of 0.64 g/g. These improvements resulted from tunable pore hydrophilicity and increased pore volumes due to altered linker lengths and building unit conformations. While this AI‐driven approach demonstrates significant advancements in MOF design,^[^ ^119^ ^]^ it also highlights challenges in synthesis scalability and cost.

Besides the chemical interactions induced by linkers, their molecular length significantly influences pore size, thereby controlling adsorption mechanisms and maximum water uptake capacity.^[^ ^120^ ^]^ In this regard, Hanikel et al.^[^ ^121^ ^]^ developed a linker extension strategy and applied (E)‐5‐(2‐carboxyinyl)‐1H‐pyrazole‐3‐carboxylic acid (H_2_PZVDC) on AlO_6_ rods, which played the pivotal role of enlarging the cage for improving water uptake, as these cages can accumulate more water molecules. This approach enlarged the MOF's cage structure, allowing for the accumulation of more water molecules, which increased water uptake by 50% compared to its parent MOF‐303 at 26% RH.^[^ ^122^ ^]^ Additionally, the study reported a reduction in heat release during adsorption, indicating a lower energy requirement for water uptake, which enhances efficiency and reversibility. The material also demonstrated exceptional stability, with only 5% capacity loss after 75 adsorption‐desorption cycles, making it highly durable for long‐term use. All of the obtained features introduce an efficient material for long‐term utilization in arid areas.

#### 3.3.3 Impact of Secondary Building Units (SBU)

The geometric and chemical attributes of SBUs and linkers play a crucial role in determining MOF framework topology, enabling the design of highly porous and structurally robust materials.^[^ ^123^ ^]^ Heavy, highly charged metal ions, such as zirconium (Zr), form strong metal‐linker bonds, resulting in MOFs with high stability in water. However, the high atomic weight of these metals negatively impacts gravimetric uptake, which refers to the amount of adsorbate a material can hold relative to its weight, typically measured in g/g.^[^ ^124^ ^]^ To improve gravimetric uptake, lighter elements such as aluminum (Al), titanium (Ti), and iron (Fe) are preferred. Additionally, bridging hydroxyl (OH) groups within SBUs serve as primary adsorption sites, commonly found in Zr_6_O_8_‐based and rod‐like SBUs, further enhancing water adsorption capacity.

**Figure 5:**
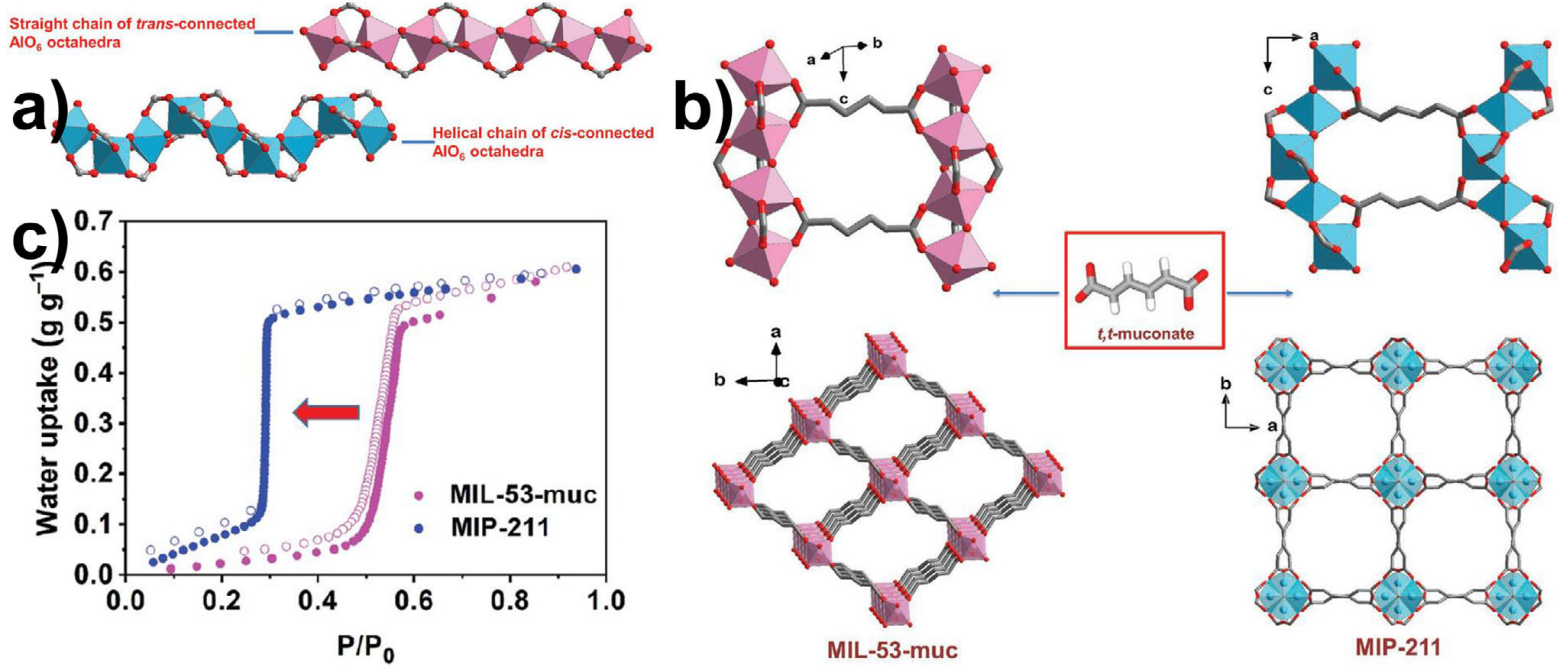
a) Corner blocks of AlO_6_ octahedra with trans‐positioned or cis‐positioned in straight or helical structures, respectively; b) comparative demonstrations of straight and helical corner structures of Al‐O chains bridged by t,t‐muconate linker resulted in two distinctive MOF‐architectures of MIL‐53‐muc (pink) and MIP‐211(blue) respectively; c) water uptake diagrams versus RH of MIL‐53‐muc and MIP‐211 (Reproduced with permission.^[^ ^125^ ^]^ Copyright 2024, Wiley).

With more precise insight, Motemp Ma Ntep et al.^[^ ^125^ ^]^ investigated how the polymorphism of SBU in MOFs influences water harvesting performance. In this context, they showcase that the replacement of the helical chain of cis‐connected AlO_6_ octahedra instead of the straight chain of trans‐connected AlO_6_ octahedra in MIL‐53‐muc renders the resultant MOF (MIL‐211) with a different topology framework as depicted in **Figure** **5**a,b. Such a minute change in the conformation of the chain leads to a more hydrophilic structure and shifts the step position of water adsorption isotherm from P/P_0_ = 0.5 in MIL‐53‐muc to P/P_0_ = 0.3 in MIL‐211, as shown in Figure 5c.

Beyond their physical properties and structural diversity, the toxicology of metals used in MOFs must also be considered, as partial MOF dissolution can contaminate the harvested water.^[^ ^126^ ^]^ To ensure safe water collection, it is preferable to use low‐toxicity linkers and metals with a low lethal dose (LD₅₀). Examples include terephthalic acid (H₂BDC), 1,3,5‐benzene tricarboxylic acid (H₃BTC), and metals such as Al, Ti, and Fe. These materials not only support efficient water adsorption but also ensure non‐toxic and safe water harvesting.

#### 3.3.4 Impact of Defects and Crystal Quality

Defect structures in solid materials significantly influence their physical and chemical properties, directly affecting AWH efficiency. These structural imperfections impact moisture adsorption, desorption rates, and active site formation, thereby altering water capture and release performance. Perfect MOF structures, with ideal crystal arrangements, are theoretically possible but practically unachievable.^[^ ^127^ ^]^ Real crystal structures always deviate from the perfect or ideal structures due to structural defects.^[^ ^128^ ^]^ Certain defect structures, such as unsaturated metal ions and uncoordinated ligands, act as acidic or basic sites, increasing the number of active adsorption sites.^[^ ^129^ ^]^ Therefore, defect engineering in MOFs plays a crucial role in optimizing structure‐property relationships for targeted AWH applications.^[^ ^130^ ^]^ This strategy enhances water adsorption capacity and stability, improving overall MOF efficiency in water harvesting.

**Figure 6:**
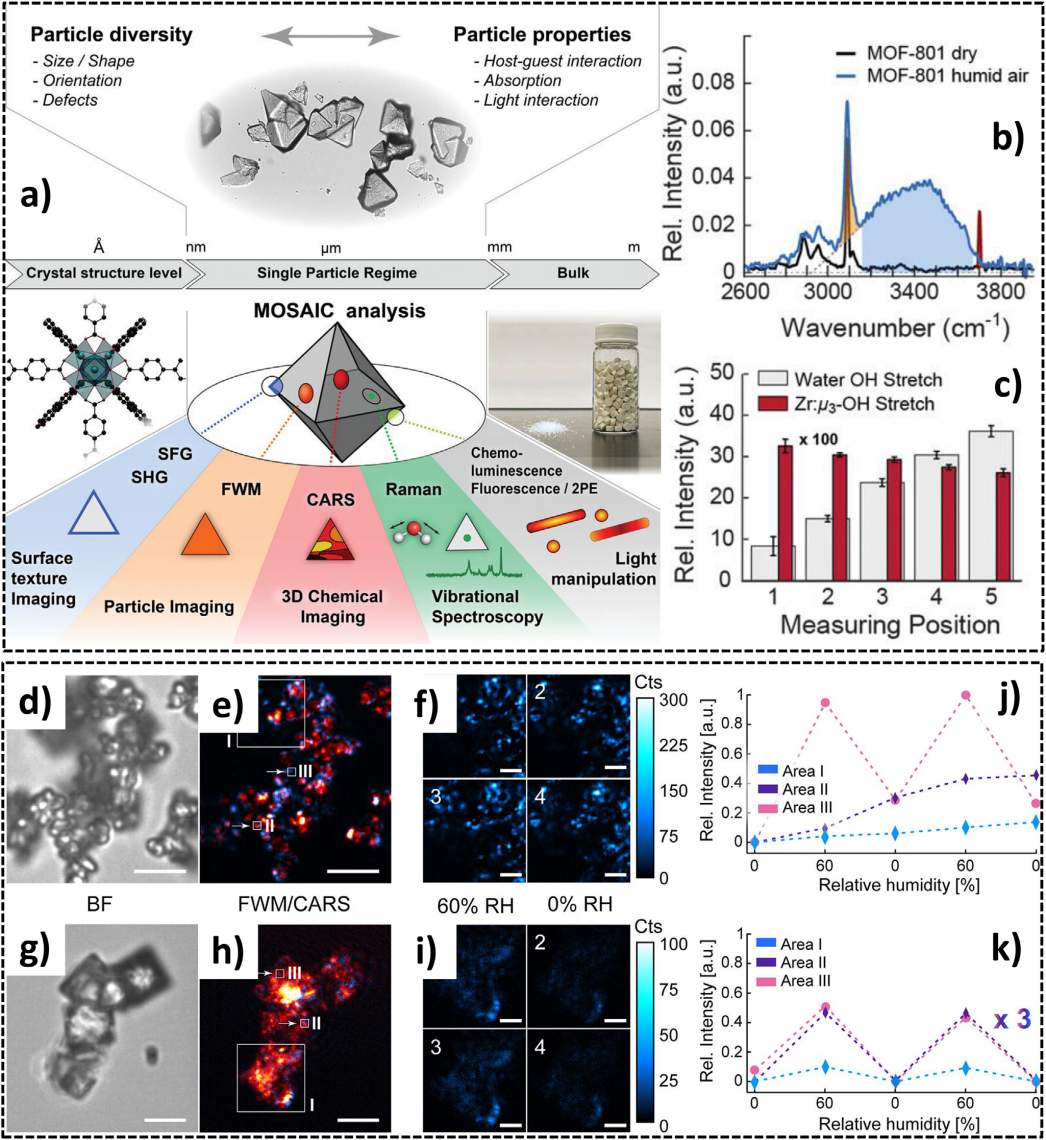
a) MOSAIC analysis of MOF‐801, illustrating structural diversity from crystal to bulk material using SFG, SHG, FWM, CARS, and Raman spectroscopy; b) Raman spectra comparing dry and humid conditions, highlighting water and Zr‐µ3‐OH stretch vibrations; c) Spatial variation of water OH stretch and Zr‐µ3‐OH stretch within the MOF‐801 crystal. (Reproduced with permission.^[^ ^131^ ^]^ Copyright 2022, Wiley); d) BF microscopy image of particle morphology; e) FWM/CARS imaging of water and open metal site distribution; f) CARS images at 60% and 0% RH showing water distribution. g) BF image of polycrystalline structures; h) FWM/CARS imaging of water uptake in defect‐rich regions; i) CARS images showing water redistribution under cyclic humidity; j,k) Quantitative plots of water content across different crystalline regions (Reproduced with permission.^[^ ^132^ ^]^ Copyright 2023, American Chemical Society).

Recent progress in single‐crystal MOF analysis has provided deeper insights into water adsorption behavior. Fuchs et al.^[^ ^131^ ^]^ introduced the MOSAIC (Multimodal Optical Spectroscopy and In situ Imaging Correlating) platform, integrating advanced techniques such as Second Harmonic Generation (SHG), Sum‐Frequency Generation (SFG), Four‐Wave Mixing (FWM), and Raman spectroscopy. This approach enables a comprehensive assessment of MOFs from their crystal structure to bulk properties (**Figure** **6**a). The study focused on MOF‐801, analyzing its water adsorption behavior under varying conditions. As shown in Figure 6a, particle size, shape, orientation, and defect distribution significantly influence MOF performance. Raman spectroscopy (Figure 6b) demonstrates notable differences in water adsorption under dry and humid conditions, with shifts in the OH stretch region, indicating interactions around Zr‐µ3‐OH sites, which are crucial for water adsorption. Additionally, spatial mapping (Figure 6c) reveals that defects and structural heterogeneities create variation in water adsorption across different sites within a single crystal, highlighting the need for precise defect control in MOF design for AWH applications.

Further research by Fuchs et al.^[^ ^132^ ^]^ examined single‐crystal (SC) and polycrystalline MOF‐801 (Figure 6d–k), demonstrating that defects and polycrystalline structures significantly impact water adsorption behavior. SC MOF‐801 particles exhibit uniform water uptake and release (Figure 6d–j), whereas polycrystalline, defect‐rich MOF‐801 particles show high adsorption heterogeneity. Bright‐field (BF) microscopy images (Figure 6d,g) reveal structural differences between SC and polycrystalline particles. FWM/Coherent Anti‐Stokes Raman Scattering (CARS) imaging (Figure 6e,h) highlights the spatial distribution of water and open metal sites, demonstrating homogeneous water distribution in SCs but highly concentrated adsorption in defect‐rich regions of polycrystalline particles. Additionally, CARS imaging under different RH conditions (60% and 0%) (Figure 6f,i) shows that polycrystalline MOFs retain more water, often failing to fully release it during desorption. This behavior is quantified in Figure 6j,k, where the relative water content across different MOF regions is plotted, emphasizing the impact of humidity on water uptake dynamics and the heterogeneity in adsorption among crystalline regions.

These findings indicate that while SC MOFs provide predictable and efficient AWH performance, defects and polycrystalline structures can lead to uneven water distribution, affecting overall efficiency and long‐term stability. The study underscores the importance of defect control and crystal quality optimization when designing MOFs for AWH applications.

### 3.4 MOF Hybrid Composites: Non‐Monolith Architectures

Beyond various modification strategies, cutting‐edge approaches are emerging to optimize MOF‐based AWH systems for improved functionality. One promising strategy is the use of composite sorbents, where hygroscopic salts are encapsulated within a MOF's porous framework.^[^ ^133^ ^]^ This approach not only mitigates the limitations of chemisorptive salts—such as corrosion, agglomeration, and deliquescence—but also enhances water uptake via synergistic interactions in salt@MOF composites.

**Figure 7:**
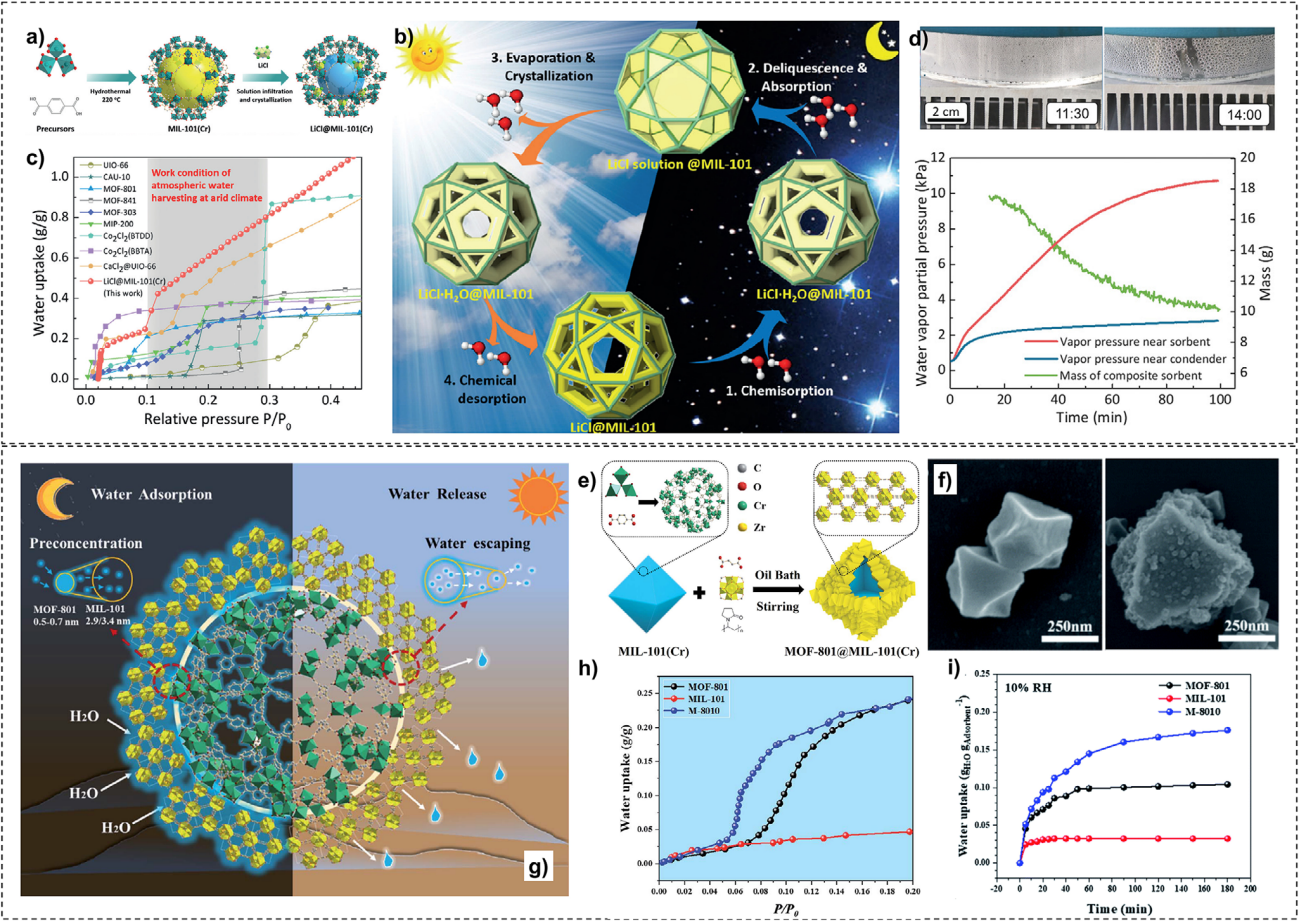
a) Stages and conditions under which composite sorbent of LiCl@MIL‐101(Cr) is produced; b) schematic illustration of complete nocturnal sorption followed by diurnal desorption cycle of LiCl@MIL‐101(Cr) over a day; c) water sorption behavior of LiCl@MIL‐101(Cr) in comparison with other MOFs and composite sorbents (Reproduced with permission.^[^ ^134^ ^]^ Copyright 2020, Wiley). Schematic illustration of e) the self‐assembly process and f) SEM images of MOF‐801@MIL‐101(Cr) supra‐particles (M‐8010) and the chemical structures of its components; g) the sorption‐desorption mechanism of M‐8010 in a 24‐hour cycle; h) the water adsorption behavior of MOF‐801 and MIL‐101 compared with their core‐shell composition (MOF‐8010) in different relative ambient water vapor pressures, and i) the time‐dependent water adsorption behavior of MOF‐801, MIL‐101, and MOF‐8010 at 10% RH (Reproduced with permission.^[^ ^135^ ^]^ Copyright 2022, Royal Society of Chemistry).

For this purpose, Xu et al.^[^ ^134^ ^]^ designed a salt@MOF material comprised of LiCl as the hygroscopic salt and within a Cr‐based MOF (MIL‐101), as shown in **Figure** **7**a. The cyclic sorption mechanism of this composite is depicted in Figure 7b, highlighting the key roles of chemisorption and encapsulation in its operation. The high pore volume (2.0 cm^3^ g^−1^) of MIL‐101 facilitates substantial salt loading, while its large surface area (4100 m^2^ g^−1^) provides an extensive adsorption interface. Noteworthy, MIL‐101's pore diameter (2.9–3.3 nm) is ideal for LiCl crystallization at the nanoscale. LiCl was selected due to its exceptional water uptake capacity under arid conditions. The three‐phase sorption process—comprising salt chemisorption, solid‐liquid deliquescence, and liquid solution absorption—works synergistically to enhance water capture efficiency. These results underscore the feasibility of MOF/salt composites for large‐scale AWH applications, particularly in arid regions, where natural sunlight can be leveraged without additional energy input. As shown in Figure 7c, these findings position MOF/salt composites as highly promising alternatives to simple MOFs for AWH. This composite enabled the development of a solar‐powered device, achieving 7.0 g/g water harvesting per cycle under laboratory conditions (1 sun radiation) and 4.5 kg/kg under natural sunlight (Figure 7d).

Beyond conventional MOF structures, supra‐particles—composed of single or multiple building blocks—offer tailored functionalities for various applications.^[^ ^136^ ^]^ In the context of AWH, core‐shell supra‐particles have emerged as practical alternatives.^[^ ^137^ ^]^ Inspired by this concept, Hu et al.^[^ ^135^ ^]^ designed a MOF@MOF core‐shell supra‐particle using MOF‐801 as the shell and MIL‐101(Cr) as the core (termed M‐8010). The synthesis process is schematically illustrated in Figure 7e, with SEM images of the particles shown in Figure 7f. During water harvesting, this structure experiences a humidity gradient from the shell toward the core (Figure 7g), which acts as a driving force for water molecules, directing them toward MIL‐101. The MOF‐801 shell preconcentrates moisture due to its hydrophilic small pores, enhancing water uptake at low humidity levels. This process increases local humidity around the MIL‐101 core, enabling it to utilize its high‐water adsorption capacity even at ultralow humidity (<10%), making it ideal for desert‐like conditions. As shown in Figure 7h, M‐8010 supra‐particles achieve a water sorption capacity of 0.18 g/g at 10% RH, which is 1.8 times higher than pristine MOF‐801 and 6 times higher than MIL‐101 under the same conditions. Not only is the water uptake higher but Figure 7i also shows that supra‐particles exhibit faster sorption kinetics. These results highlight the potential of core‐shell MOF structures in enhancing water capture efficiency for AWH applications.

Other MOF‐based sorbents for AWH systems, featuring varied desorption strategies, are summarized in **Table** **3**. However, despite their impressive advancements in enhancing water uptake characteristics, especially at low RH, the powdery format of MOFs limits their performance and scalability.

**Table 3:** Recent MOF‐based water sorbents with different adsorption properties.

MOF‐based Sorbent Components	Water Uptake Capacity / Productivity	RH [%]	BET Surface Area [m^2^ g^−1^]	Pore Volume [cm^3^ g^−1^]	Pore Size [nm]	3D Structure	Refs.
UiO‐66‐NH_2_, Carbon Black, LiCl,	0.57 g/g 1.44 g/g 1.86 g/g	30 60 90	–	–	–	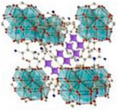	[138]
UiO‐66, MIL (101)(Cr)	0.408 g/g	30	1381	1.29	–	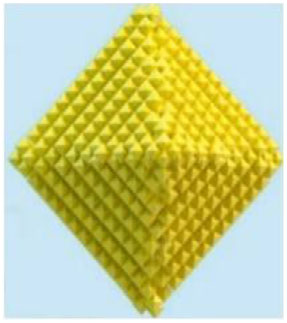	[139]
MIL‐96 (Al), MIL‐100 (Fe), Chitosan‐modified glass fiber, MWCNT	0.33 g/g 0.58 g/g 0.70 g/g	30 60 90	1535.28	0.76	–	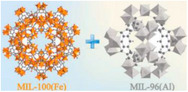	[140]
MIL‐101(Cr), LiCl	0.77 g/g	30	1179	0.69	–	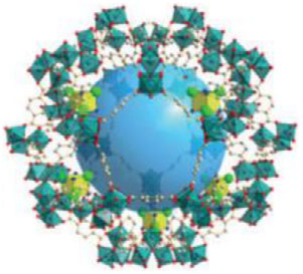	[134]
MIL‐101(Cr), Melamine Sponge	1.232 g/g	70	–	–	–	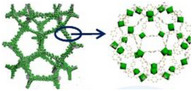	[141]
MIL‐101‐Cr (HF)	3.2 mL/g_MOF_.day	80	–	1.4	1.5‐2.5	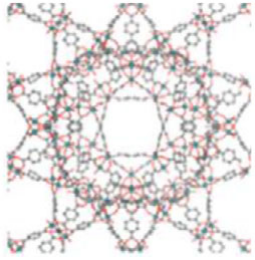	[142]
MIL‐160(Al), MOF‐303	0.44 g/g 0.46 g/g 0.56 g/g	30 60 90	917.59	0.44	0.7	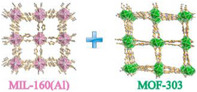	[143]
MOF‐303	1.3 L/kg.day	32	–	–	–	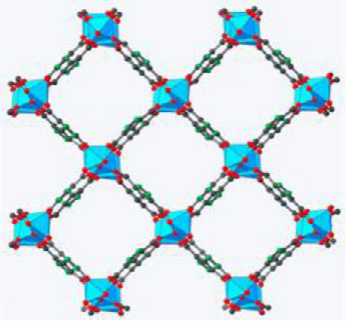	[144]
MOF‐LA2‐1	0.68 g/g	26	1892	0.67	–	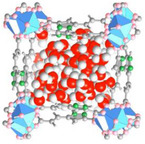	[121]
MOF‐801	2.8 L/kg.day	20	–	–	–	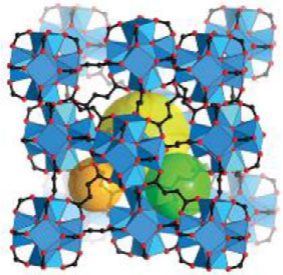	[145]
N‐doped MOF‐801	0.29 g/g	30	497.5	0.23	64‐118	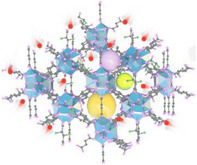	[146]
MOF‐801, P(NIPAM‐GMA)	0.33 g/g (1.6 kg/kg.day)	20	–	–	–	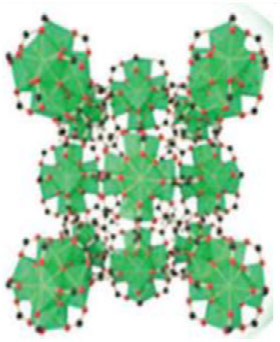	[147]
MOF‐801, MIL‐101(Cr)	0.148 0.180 0.220	8 10 15	928.2	0.76	–	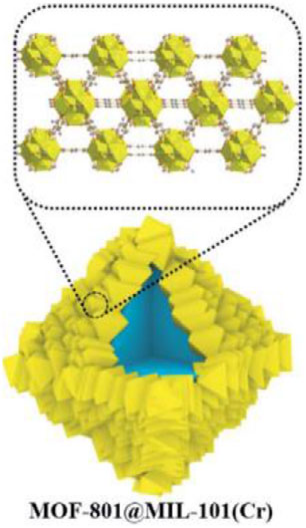	[135]
MOF‐808, CaCl_2_	0.56	30	348	0.07	–	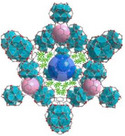	[148]
MOF‐808‐Cl, MOF‐808‐Br, MOF‐808‐I	0.65 g/g 0.38 g/g	30 5	1940 1690 1500	–	–	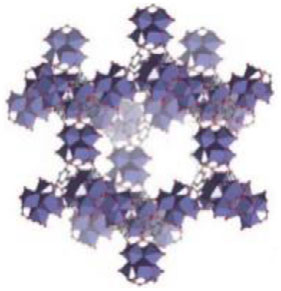	[107]
AlCl_3_‐Fuma MOF, TiO_2_	130 mg/g	20	–	–	–	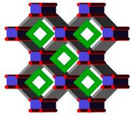	[149]
CuBTC, Aminoclay, Graphene Oxide	0.431 g/g	90	1569	–	–	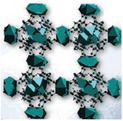	[150]
NO‐PI‐3‐COF	0.15 g/g 0.27 g/g	21 95	–	–	2.1	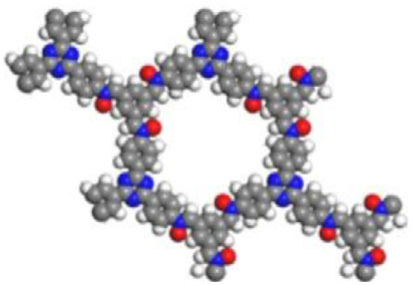	[151]
BMOF‐3 (Al‐furmarate, MIL‐88A)	0.47 g/g 0.48 g/g 0.56 g/g	30‐90	1202.99	0.51	1.7	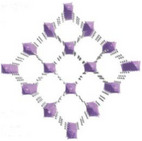	[152]
Cu‐AD‐SA	0.13 g/g 0.16 g/g	5.3 20	651	0.34	50	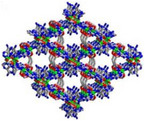	[153]
CAU‐10‐H	0.32 g/g	18	–	–	–	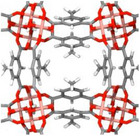	[154]
CAU‐250 (Calcined @ 250°)	0.48 g/g	40	1583	0.75	0.66	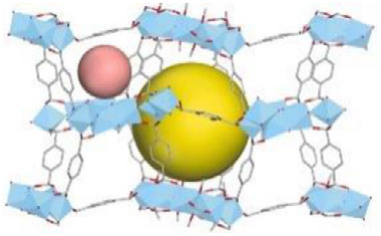	[155]
AlPO‐18	0.30 g/g 0.42 g/g	13 100	656	0.31	–	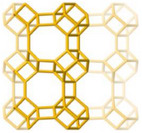	[156]
Cr‐soc‐MOF‐1	1.95 g/g	75	–	–	–	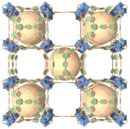	[157]
ZrMOF‐1 ZrMOF‐2	0.80 g/g 0.83 g/g ‐	60 90 ‐	1877 553	0.73 0.22	154‐218 220‐285	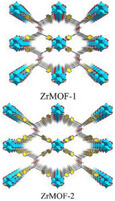	[158]
COF‐432	0.30 g/g 0.23 g/g	95 20‐40	895	0.43	80	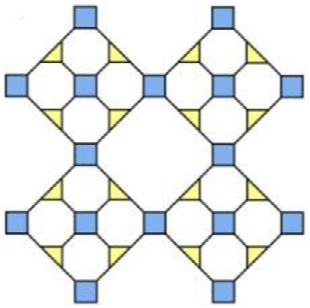	[108]
Pyrene‐based COF	311 cm3/g	90	705	–	0.75	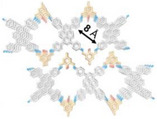	[159]
TpPa‐1 COF, LiCl	0.37 g/g 0.80 g/g	30 90	357	–	1.26	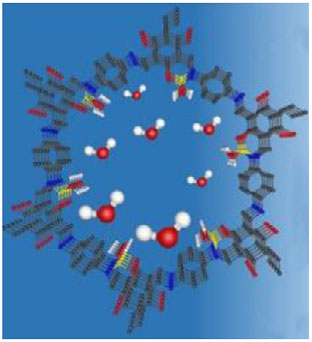	[160]
Ppy‐based COF, LiCl	0.77 g/g 2.56 g/g	30 80	298	–	450‐1.7	–	[161]
COF‐v, PAA	302.8 mg/g 907.2 mg/g	70 90	6.358	–	3.26 – 11.37	–	[162]
MIL‐100(Fe), Silica gel	0.351 kg/kg	22‐56	1179.1826	0.60796	–	–	[163]
MIL‐100(Fe), LiCl MIL‐100(Fe), CaCl_2_ MIL‐100(Fe), MgCl_2_	1.75 g/g 1.03 g/g 0.53 g/g	65 65 65	251.9844 254.7719 50.5118	0.070455 0.076419 0.138035	2.5824 2.6172 11.2362	–	[164]
MIL‐100(Fe), MgCl_2_	0.533 g/g 1.062 g/g	35 80	–	–	–	–	[165]
MIL‐101(Cr), CaCl_2_	0.59 g/g	31.3	–	–	–	–	[166]
MIL‐101(Cr), PDMAPS, LiCl	0.614 g/g 1.827 g/g	40 90	–	–	–	–	[167]
Al‐MOF, Cu_x_S‐Cu	0.161 g/g	30	376.38	0.1487	–	–	[168]
MOF‐5, GO	542 mg/g 1137 mg/g	55 75	–	–	–	–	[169]
Co‐MOF‐31	0.79 g/g 0.98 g/g	30 98	1619.3	–	–	–	[170]
Carbonized Ca‐MOF (PCC‐42)	0.45 g/g 0.52 g/g 0.76 g/g 0.90 g/g	20 30 40 50	692	0.86	–	–	[171]
MOF‐303	0.30 g/g 0.63 g/g	15 95	>1300	–	–	–	[172]
MOF‐801	0.26 g/g	30	997.8	0.234	92‐197nm	–	[173]
MOF‐801, CNT	0.219 kg/kg	20	354.77	0.0842	4.26	–	[174]
Ni_2_Cl_2_BTDD	0.53 g/g	30	1554	–	–	–	[175]
Ni_2_F_2_BTDD Ni_2_Cl_2_BTDD Ni_2_Br_2_BTDD	< 1 g/g < 1 g/g 0.64 g/g	32 32 25	–	–	–	–	[176]
LiMOF‐derived porous carbon	1.34 g/g 1.69 g/g 2.56 g/g 4.23 g/g	20 40 60 80	173.2	0.29	–	–	[177]
ZIF‐8‐derived nanoporous carbon	380 mg/g	40	1481	–	–	–	[178]

## 4 Hygroscopic Highly Porous Monolith with Microporous Structures for AWH

### 4.1 Porous Monolith: Definitions and Background

Unlike highly crystalline and microporous MOFs, porous monoliths are bulk materials with interconnected pore networks, offering tunable porosity and high surface area. Porous monoliths exhibit pore sizes ranging in micropore scales (from 2 nm to a few hundred µm), facilitating efficient vapor transport and capillary‐driven water absorption.^[^ ^179^ ^]^ Their ultralow density (high porosity),^[^ ^180^ ^]^ high surface area, and mostly open‐cell structure facilitate efficient mass transfer between pores, making them promising materials for AWH.^[^ ^181^ ^]^ These materials include foams, aerogels, xerogels, and cryogels, each distinguished by their fabrication method and the resulting structure. Foams, with porosities ranging from 50% to 75%, are produced via polymerization, phase separation, or templating, forming lightweight structures with controlled pore sizes.^[^ ^182^, ^183^ ^]^ Aerogels, with ultrahigh porosities (90%–99.9%), are synthesized through the sol‐gel process, followed by supercritical drying,^[^ ^184^ ^]^ which removes the solvent under supercritical conditions to preserve their open‐cell network.^[^ ^185^ ^]^ Xerogels, with porosities between 80% and 95%, undergo ambient drying, where capillary forces induce partial shrinkage, increasing density.^[^ ^186^ ^]^ Cryogels, with porosities typically between 85% and 99%, are fabricated via freeze‐drying, where ice crystal templating forms an interconnected macroporous network.^[^ ^187^ ^]^

Although foam, aerogels, cryogels, and xerogels differ in fabrication and structure, they share fundamental properties that enable efficient water vapor sorption and transport. In this work, the term “monolith” is used to describe these materials collectively, emphasizing their interconnected porosity and potential for AWH applications. However, for specific examples, the exact term used in the references are used to avoid confusion. The performance of these monoliths is highly dependent on the choice of precursor materials, which dictate their porosity, mechanical stability, and surface chemistry. Silica‐based,^[^ ^188^ ^]^ synthetic‐based (e.g., polyimide,^[^ ^189^ ^]^ polyvinyl alcohol,^[^ ^190^ ^]^ polyurea,^[^ ^191^ ^]^ resorcinol‐formaldehyde,^[^ ^192^ ^]^ melamine‐formaldehyde,^[^ ^193^ ^]^ polyurethane,^[^ ^194^ ^]^ etc.^[^ ^195^ ^]^), bio‐based (e.g., cellulose,^[^ ^196^, ^197^, ^198^ ^]^ chitosan,^[^ ^199^ ^]^ wood),^[^ ^200^ ^]^ and carbon‐based monoliths (e.g., pyrolyzed materials,^[^ ^201^ ^]^ carbon black,^[^ ^202^ ^]^ graphene,^[^ ^203^ ^]^ carbon nanotube)^[^ ^204^ ^]^ are among the most relevant for AWH due to their high porosity and tunable surface properties.^[^ ^184^ ^]^

### 4.2 Mechanisms, Chemistry, and Notable Examples of Monolith‐Based AWH

Monolith's 3D porosity results in an exceptional surface area, providing abundant adsorption sites and significantly increasing sorption capacity.^[^ ^205^ ^]^ Their interconnected pore network also enhances water transportation^[^ ^206^ ^]^ as well as improve water desorption efficiency.^[^ ^207^ ^]^ The efficiency of monolith in AWH can be explained through thermodynamic principles. Water sorption occurs when Gibbs free energy, ΔG, is negative, influenced by ΔH and ΔS: Δ𝐺 = Δ𝐻 ‐ TΔ𝑆. The hydrophilic surface of monoliths promotes exothermic interactions, favoring sorption. However, as water molecules enter the confined monolith pores, entropy decreases. Despite this, sorption remains thermodynamically favorable due to the strong enthalpic interactions. During desorption, solar heating raises the temperature (T), shifting ΔG to negative values, and facilitating water release.^[^ ^208^ ^]^

Furthermore, the Kelvin equation, which relates the vapor pressure of water in a porous material to the curvature of the liquid meniscus, can be applied to describe the sorption and desorption processes in monoliths: ln(P/P_0_)= 2γV_m_/rRT, where 𝑃 is the vapor pressure, 𝑃_0_​ is the saturation vapor pressure, γ is the surface tension, 𝑉_𝑚_ is the molar volume of liquid (or water), 𝑟 is the pore radius, 𝑅 is the gas constant, and 𝑇 is the temperature.^[^ ^209^ ^]^ The optimal pore size distribution is 10‐100 nm for AWH based on capillary forces and the Kelvin equation. Therefore, the small pore size of monoliths contributes to higher vapor pressure differentials, enhancing water vapor adsorption at lower humidity. These characteristics make monoliths highly suitable for AWH applications.^[^ ^210^ ^]^

**Figure 8:**
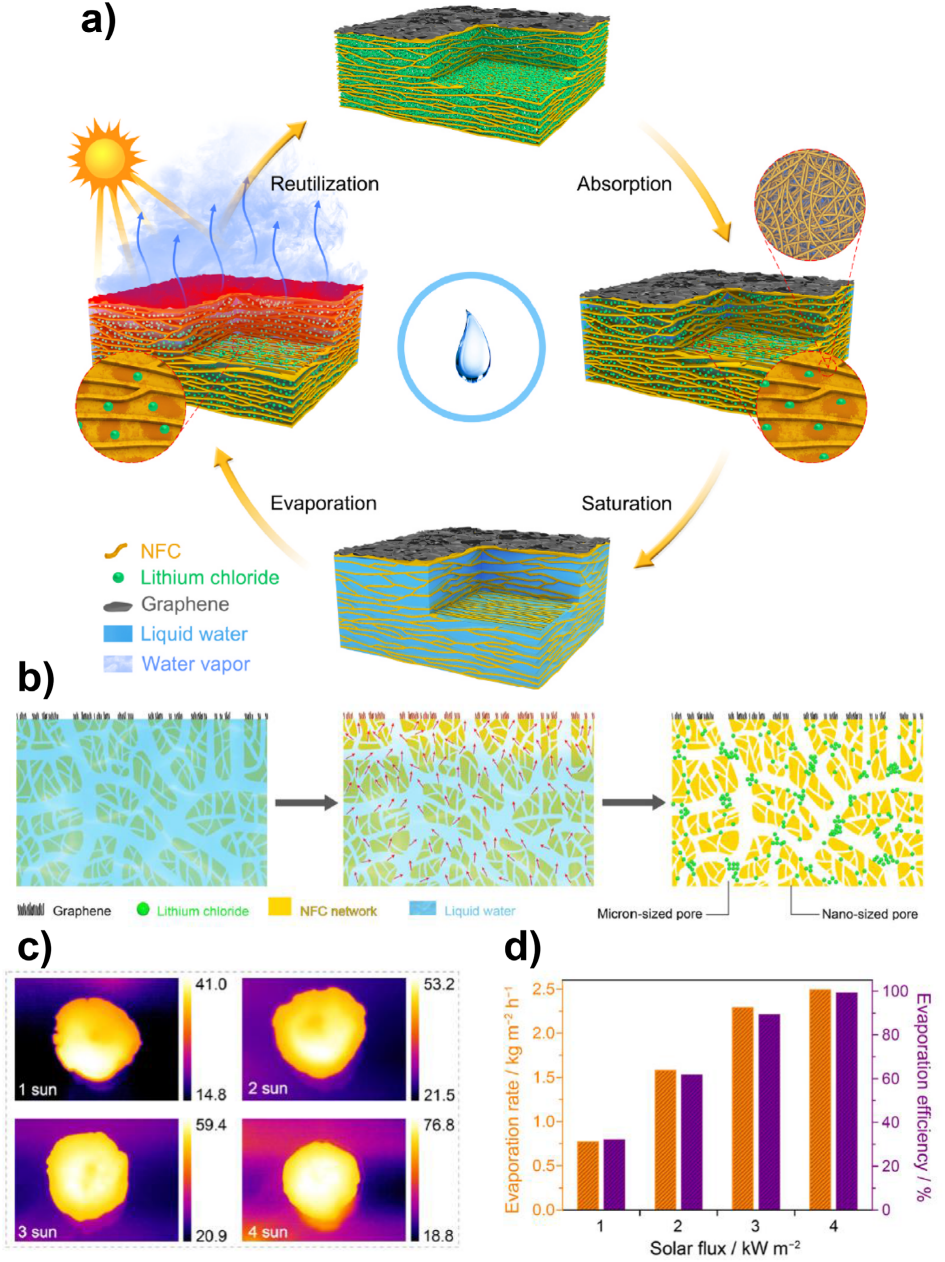
a) Schematic illustration of different stages of the AWH process of LiCl‐incorporated nano‐fibril‐cellulose‐derived monolith; b) water transportation during solar‐powered desorption in a hierarchical‐designed monolith; c) IR images of the monoliths’ top surface in varied solar intensities after 1000 s and d) their corresponding evaporation rate and efficiency (Reproduced with permission.^[^ ^212^ ^]^ Copyright 2021, Elsevier).

While monoliths offer high porosity and excellent water sorption properties, their performance can be further enhanced by integrating hygroscopic salts. These salts exhibit high water uptake even at low humidity, but their practical application is hindered by leakage, corrosion, and agglomeration.^[^ ^211^ ^]^ To address this, Wang et al.^[^ ^212^ ^]^ introduced a system based on nano‐fibrillated cellulose (NFC) integrated with LiCl and graphene as a solar absorber to develop a solar‐powered nanostructured biopolymer hygroscopic bilayer aerogel, as depicted in **Figure** **8**a. In this work, the abundant hydroxyl groups of cellulose nanofibrils endowed the formulation with superb hydrophilicity to adsorb water molecules, while LiCl particles were trapped within the aerogle's pores. The process involves atmospheric water being adsorbed until LiCl reaches its saturation point. From a topological perspective, aerogel's porosity not only helps retain LiCl but also facilitates water transfer toward the graphene‐based layer through capillary forces induced by the interconnected network, as shown in Figure 8b. Here, the water is exposed to heat generated from light‐heat conversion (Figure 8c). This synergistic design resulted in a high adsorption capacity of 0.55 g/g at ∼18% RH under natural sunlight (0.1–0.56 kW m^−^^2^), with performance further improving under multiple sunlight intensities, as shown in Figure 8d.

**Figure 9:**
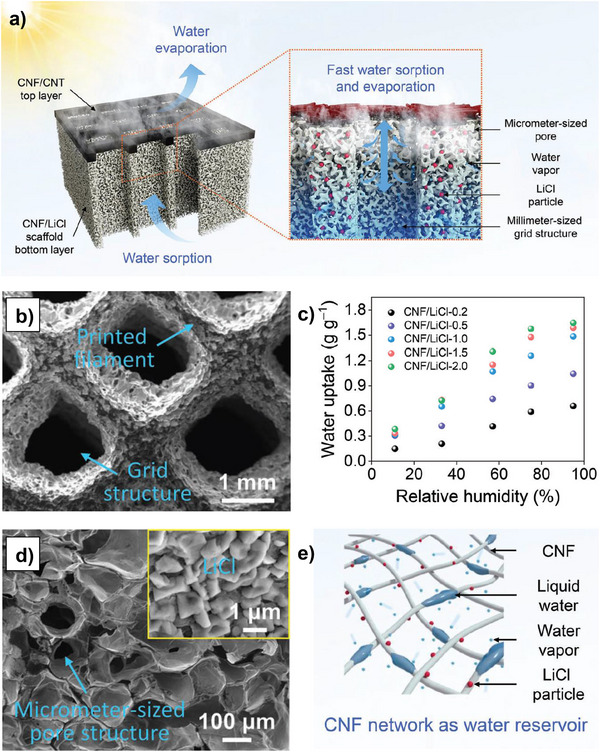
a) Schematic illustration of the 3D printed bilayer hierarchically designed CNF monolith sorbent impregnated with LiCl; SEM images of b) the top view; c) the water uptake capacity of the sample in varied RH, and d) the microstructure of micrometer‐sized pore structure located in a longitudinal direction filled with LiCl particles (Reproduced with permission.^[^ ^213^ ^]^ Copyright 2024, Wiley).

Scaling AWH systems from laboratory to real‐world applications requires optimizing macroscale and microscale designs. Additive manufacturing (3D printing) is revolutionizing AWH by enabling precise fabrication of complex, tailored structures that enhance efficiency and durability. The ability to combine different materials in a single structure allows researchers to develop next‐generation AWH devices, improving performance, adaptability, and integration into existing infrastructure. In this regard, Zhu et al.^[^ ^213^ ^]^ employed 3D printing technology to create a novel hierarchical monolith with a grid‐like structure, as depicted in **Figure** **9**a. The millimeter‐scale grid structure enhances mass transfer, improving air circulation and water vapor interaction (Figure 9b,c). This design maximizes water capture efficiency across a wide humidity range (Figure 9d). The sorption layer, composed of cellulose nanofiber (Tempo‐CNF) and LiCl, enables moisture absorption even under arid conditions, making it highly suitable for semi‐arid regions where conventional methods are less effective, as illustrated in Figure 9e. Beyond water capture, Zhu et al. also integrated a photothermal absorber layer, consisting of CNF and carbon nanotubes (CNTs), to enhance light absorption and localized heating (Figure 9a). This photothermal effect accelerates water evaporation, improving monolith regeneration for continuous harvesting cycles. These innovations underscore the potential of 3D printing in AWH, enabling precise control over monolith structure and composition. While promising, current designs still require optimization to reduce sorption time and energy demands for desorption. Future advancements may focus on integrating cellulose, hygroscopic salts, and MOFs into printed structures, further enhancing AWH efficiency and sustainability.

**Figure 10:**
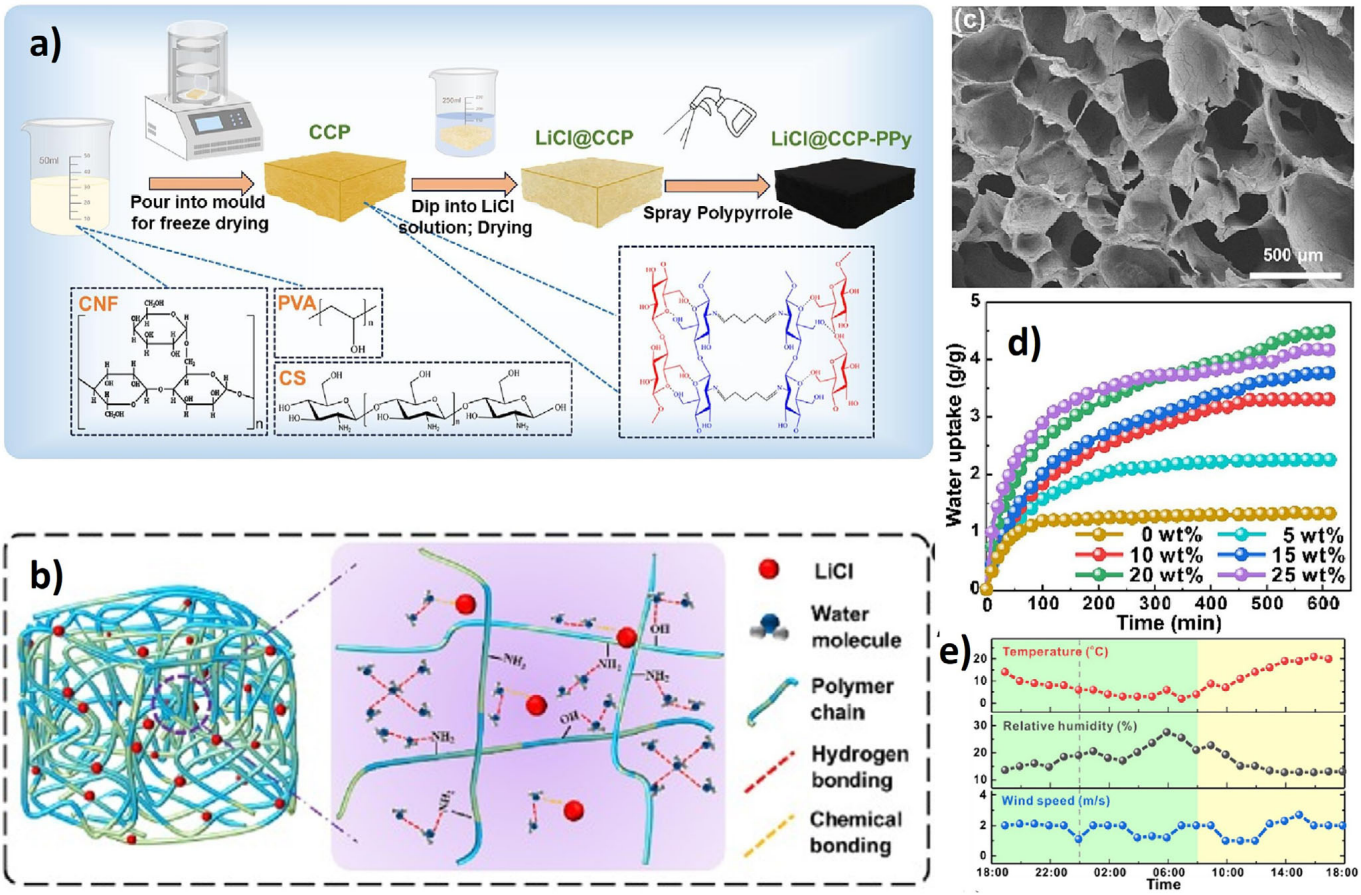
a) Fabrication steps of LiCl@CCP‐PPy composite monolith; b) Illustration of the role of hydrophilic functional groups in water adsorption, where hydroxyl and amino groups facilitate multilayer physical adsorption; c) SEM image representation of the microporous structure of LiCl@CCP‐PPy; d) Real‐world environmental testing, depicting recorded variations in temperature, humidity, and wind speed (Reproduced with permission.^[^ ^214^ ^]^ Copyright 2024, Elsevier).

One of the key challenges in bilayer monoliths is efficiently delivering stored water to the top layer for evaporation and desorption. While the high porosity of monoliths enhances water uptake, it also introduces a tortuosity effect, which hinders desorption rates by prolonging the water path to the solar‐powered heating layer. Addressing this requires breakthroughs in porosity design to improve water transport efficiency. Li et al.^[^ ^214^ ^]^ developed a composite porous polymeric foam (LiCl@CCP‐PPy) for AWH, optimizing its efficiency in low‐humidity environments. The synthesis of LiCl@CCP‐PPy, as shown in **Figure** **10**a, involved embedding LiCl into a polymeric matrix composed of cellulose nanofibers (CNF), chitosan (CS), and polyvinyl alcohol (PVA), providing a high surface area and strong hydrophilicity. The addition of polypyrrole (PPy) facilitated water desorption in the solar evaporation process. As shown in Figure 10b, the presence of numerous functional groups, such as hydroxyl and amino groups, on the polymeric chains of the composite foam imparts strong hydrophilic properties. Hydration occurs when LiCl adsorbs water from the air, triggering multilayer physical adsorption as captured water molecules attract additional moisture. Beyond its excellent hydrophilicity, the micron‐sized pores in the LiCl@CCP‐PPy monolith further enhance water transport (Figure 10c), improving mass transfer efficiency. A key feature of this study is the integration of LiCl within the polymer matrix, preventing salt leakage while maintaining high moisture absorption. As the salt content increased, water uptake also improved, reaching a maximum absorption capacity of 4.48 g g^−1^ under controlled conditions (25 °C, 90% RH), outperforming conventional hygroscopic materials (Figure 10d). Environmental parameters, including temperature, humidity, solar radiation flux, and wind speed, were continuously monitored in real‐world testing in the Tengger Desert, with recorded trends shown in Figure 10e. The results indicate that the desert temperature remained below 20 °C, with humidity levels below 30% RH and a maximum solar radiation flux of ≈1 kW m^−2^. Even under these low ambient temperatures and weak solar radiation, LiCl@CCP‐PPy exhibited high photothermal performance, reaching a surface temperature of 38.8 °C, achieving a water collection rate of 0.64 L m^−2^ d^−1^.

**Figure 11:**
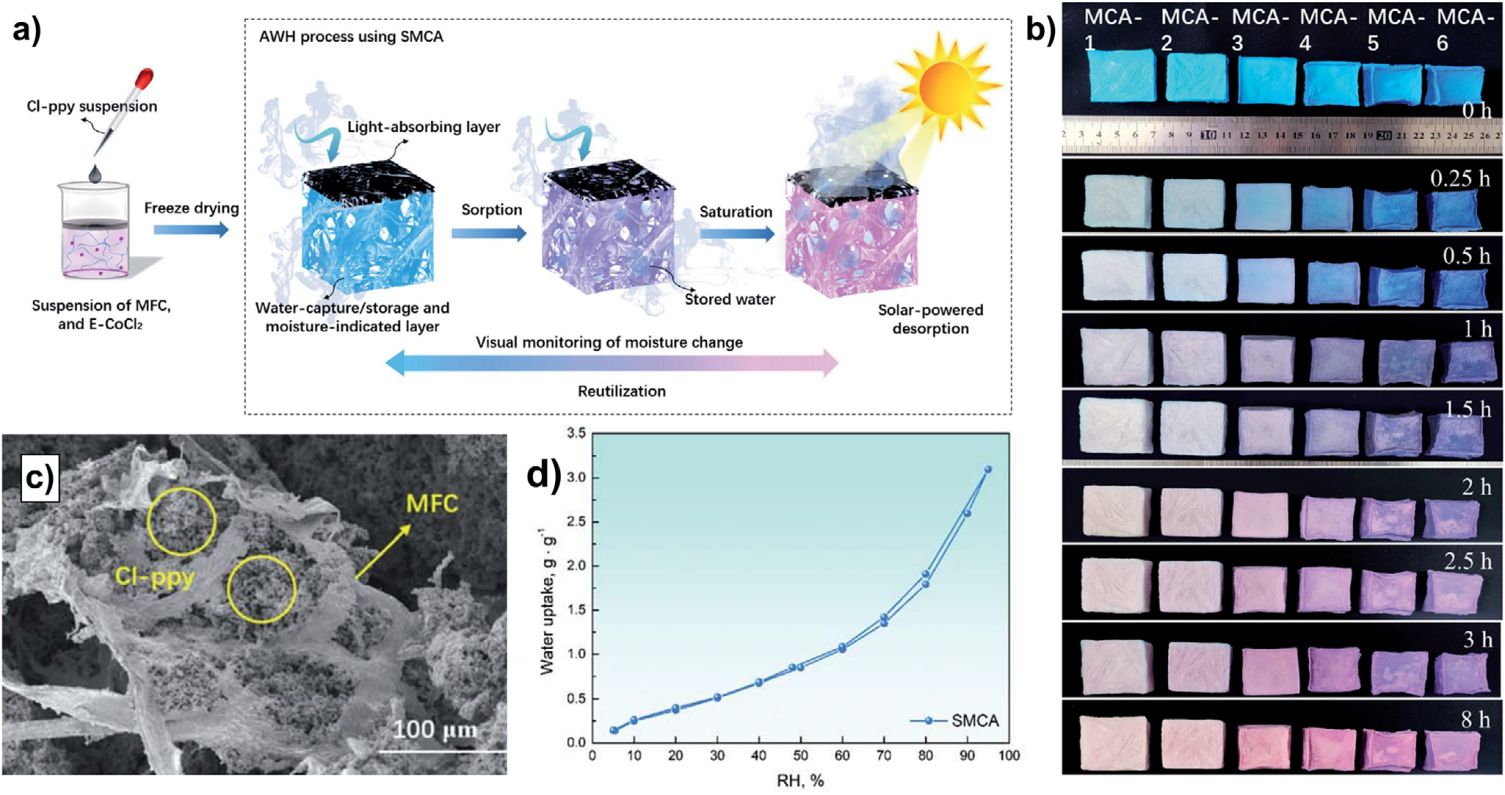
a) Preparation and AWH process of solar‐driven moisture‐indicating cellulose monolith (SMCA); b) digital images of moisture‐indicating monolith (MCA)’s color change from blue to pink proportional to absorbed moisture during water uptake at 65% RH; c) light‐absorber layer comprised of LiCl‐decorated poly‐pyrole (Cl‐ppy) and micro fibrillated cellulose (MFC); d) water uptake isotherm of SMCA (Reproduced with permission.^[^ ^215^ ^]^ Copyright 2021, Royal Society of Chemistry).

Beyond structural and physical optimizations, chemistry plays a crucial role in enhancing AWH materials. One fascinating example is the visual tracking of water adsorption, where monoliths change color based on their water content. Sun et al.^[^ ^215^ ^]^ developed a moisture‐indicating cellulose aerogel, allowing real‐time observation of water uptake, as illustrated in **Figure** **11**a. This innovative design was achieved by embedding ethanolamine‐decorated cobalt chloride (E‐CoCl₂) into microfibrillated cellulose (MFC) to form a moisture‐responsive aerogel (MCA). The monolith was prepared by mixing E‐CoCl₂ with MFC in varying concentrations (0.05–0.5 g) before freeze‐drying the samples. The resulting monoliths (MCA‐x, where x corresponds to E‐CoCl₂ content) exhibited a progressive color change from blue to purple to pink as water was adsorbed (Figure 11b). This transformation is attributed to cobalt's coordination chemistry, where water molecules alter the d‐shell electronic structure of cobalt ions, causing a visible shift in color.

To enhance both sorption and desorption, Sun et al. incorporated LiCl‐decorated polypyrrole (Cl‐ppy) solar absorber as a double‐layer system (Figure 11c). This configuration enabled efficient moisture capture and rapid solar‐driven desorption, making it highly effective across a wide humidity range (25–85%), as depicted in Figure 11d. The key innovation of this study lies in E‐CoCl₂, which simultaneously absorbs moisture and provides a visual cue for stored water content. By integrating smart, color‐responsive chemistry into functional materials, Sun et al.’s work opens new possibilities for next‐generation AWH technologies that are both efficient and visually informative.

The porous structure and engineerable nature of the monoliths make them promising candidates for AWH applications. **Table** **4** provides a list of recently developed monoliths designed for AWH. However, a significant hurdle remains: developing monolith materials that can perform effectively in regions with lower humidity. This necessitates advancements in monolith design to improve their ability to capture and release water vapor efficiently under varying environmental conditions. Also, it is crucial to ensure these materials are practical and cost‐effective for large‐scale manufacturing and deployment. Addressing these challenges through research and innovation will be vital in realizing the full potential of monolith technologies in mitigating global water scarcity issues. Therefore, we will explore monoliths combined with MOFs in the next section.

**Table 4:** Recent monolith‐based AWH systems.

Monolith skeleton	Hygroscopic agent	Water uptake capacity (g/g)	RH [%]	Photothermal material	Maximum temperature [°C]	Light intensity	Evaporation rate [kg m^−2 ^h^−1^]	Refs.
Cellulose nanofiber	LiCl	0.55 0.80 0.92 2.36	18 33 42 95	Graphene	50.7	0.1–0.56 kW m^−2^		[212]
Cellulose nanofiber	LiCl	0.39 1.65	11 95	CNT	55			[213]
Cellulose	LiCl	2.5‐16	60‐90	–	–	0.9‐1.1 sun	–	[216]
Cotton cellulose, Aramid nanofibers	NaCl	–	–	Polypyrole	67.7	1 sun	5.368	[217]
Guar gum, cellulose nanofiber	LiCl	1.94	90	CNT	57.5	1 sun	–	[218]
Cellulose microfibril, Ethanolamine‐decorated CoCl2 (E‐CoCl_2_)	LiCl	0.49 0.69 1.07 2.38	25 45 65 85	Polypyrole (ppy)	84.3	1 sun	1.391	[215]
Hydroxypropyl cellulose (HPC), PNIPAm	Ethanolamine‐decorated LiCl (E‐LiCl)	0.46 0.91 1.45 2.95	30 50 70 90	PANI	–	1 sun	1.97	[206]
Hydroxypropyl methylcellulose (HPMC), SA	LiCl	0.94 4.11	30 80	CNT	70	1000 W m^−2^	–	[219]
Cotton‐derived Carbon monolith	LiCl MgCl_2_	0.9 2.1	60 90	Graphene oxide	55.8	1.3 sun	1.63	[220]
Carbonized cellulose nanofiber	LiCl	0.93 1.57	30 50	Graphite	–	20000‐100000 Lx	–	[221]
Carbonized wood	ZnCl_2_	0.95	70	Pt‐modified g‐C3N4 nanosheets	–	1 sun	–	[222]
Carbonized loofah powder	LiCl	1.3	90	Reduced graphene oxide	70	1 sun	4.0	[223]
Carbonized resorcinol formaldehyde (RF),	–	0.02 0.38 0.47	30 60 95	–	–	–	–	[224]
Silk fibroin	Carboxymethyl Chitosan, CaCl_2_	0.27 0.66 1.35	50 70 90	Copper sulfide nanoparticles (CuSNP)	110	4 sun	–	[225]
Sodium alginate (SA)	LiCl	1.92	80	Graphene oxide	70	–	36	[226]
Sodium alginate (SA)	CaCl_2_	0.66 0.90 1.35 1.91 2.29	30 40 60 80 90	Chelation of Tannic acid & Fe^3+^	71.5	1 sun	1.77	[227]
Sodium alginate (SA), Carboxymethyle Chitosan (CCS)	CaCl_2_	0.20 0.39 1.90	25 60 90	Carbon powder	48.8	1 sun	1.4	[228]
Carbonized SA, aramid fiber	LiCl	0.48 1.15 3.29	30 60 90	Expanded graphite	75	1 sun	2.4	[229]
Loofah grafted calcium alginate	–	0.50 1.20	30 60	Carbon nanosphere	60	1 sun	1.5	[230]
Poly(vinyl alcohol) (PVA), SA	LiCl	3.4	90	CNT	71.5	1000 W m^−2^	–	[231]
Poly(vinyl alcohol) (PVA),	LiCl, MgSO_4_	0.974 1.216 1.870 4.525	30 50 70 90	Activated carbon	75	900 W m^−2^	–	[232]
Poly(vinyl alcohol) (PVA), Polyacrylamide (PAM)	LiCl	0.785 1.443 3.507	30 60 90	GO	57	1 sun	3.82	[233]
Polyacrylamide (PAM)	LiCl	1.79 2.58 3.86	30 50 70	–	–	–	–	[44]
Polyacrylamide (PAM)	LiCl	1.50	60	Carbon Cloth	75	1 kW m^−2^	–	[234]
Polyacrylamide (PAM)	LiCl	1.0 1.5 2.36 4.29 5.86	30 40 60 80 90	MXene	60.1	1 kW m^−2^	–	[235]
Polyacrylamide (PAM), chitosan	LiCl	0.78 1.36 2.32 3.89 5.00	30 40 60 80 90	Giene	57.6	1 kW m^−2^	–	[236]
Polyacrylamide (PAM)	PAM			Multi‐walled CNT (MWCNT)		1 sun	2.0	[237]
Poly(N‐isopropylacrylamide) (PNIPAAM)	LiCl	1.1 3.96	60 90	Carbon nanoparticles	53	1 sun	–	[238]
Poly(N‐isopropylacrylamide) (PNIPAAM)	CaCl_2_	1.4 3.6	60 90	Graphene oxide	40.5	1 sun	–	[239]
Poly(N–isopropylacrylamide) (PNIPAAM)	CaCl_2_	0.76 1.83 2.15 2.68 3.10	30 60 70 80 90	Polydopamine (PDA) Polypyrole (PPy)	75.9	1 sun	1.75	[240]
Poly(N‐isopropylacrylamide) (PNIPAAM)	PEG	3.2	97	Fe_3_O_4_	70.5	1 kW m^−2^	–	[241]
Poly(2‐acrylamido‐2‐methyl‐1‐propane sulfonic acid) (PAMPS)	LiCl	0.65 1.00 1.87 5.45	15 30 60 90	CNT	75	1.0 kW m^−2^	–	[242]
Sodium polyacrylate, PNIPAAm	LiCl	0.68 1.61 1.92 2.47 2.76	30 60 70 80 90	Polydopamine nanoparticles	57.2 99.3	0.6 sun 1.6 sun	–	[243]
Poly(diallyl dimethylammonium chloride) PDDA	–	0.13 0.37 1.1	30 60 90	Reduced graphene oxide	>80	1 kW m^−2^	–	[244]
PDMAPS	LiCl	1.08 1.52 2.36	30 50 70	Graphene oxide	62	1 sun	–	[245]
Holey graphene fiber	LiCl	0.20 4.14	10 90	Graphene	≈50	1 sun	–	[246]
Benincasa hispidas	–	4.83	98	Polypyrole	74.1	1 sun	2.49	[247]

## 5 Multiscale Porous Integration: MOF‐Based Monoliths for AWH

MOFs and monoliths differ in pore size and formation methods—MOFs are synthesized through precise chemical reactions, while monoliths are produced via gelation and controlled drying. Despite these differences, their porous structures allow for the creation of MOF‐based monoliths, which integrate the high surface area of MOFs with the interconnected, lightweight framework of monoliths.^[^ ^248^ ^]^ This hierarchical structure enhances water uptake and facilitates efficient desorption, making MOF‐monolith composites promising for AWH applications.^[^ ^249^, ^250^, ^251^, ^252^ ^]^

**Figure 12:**
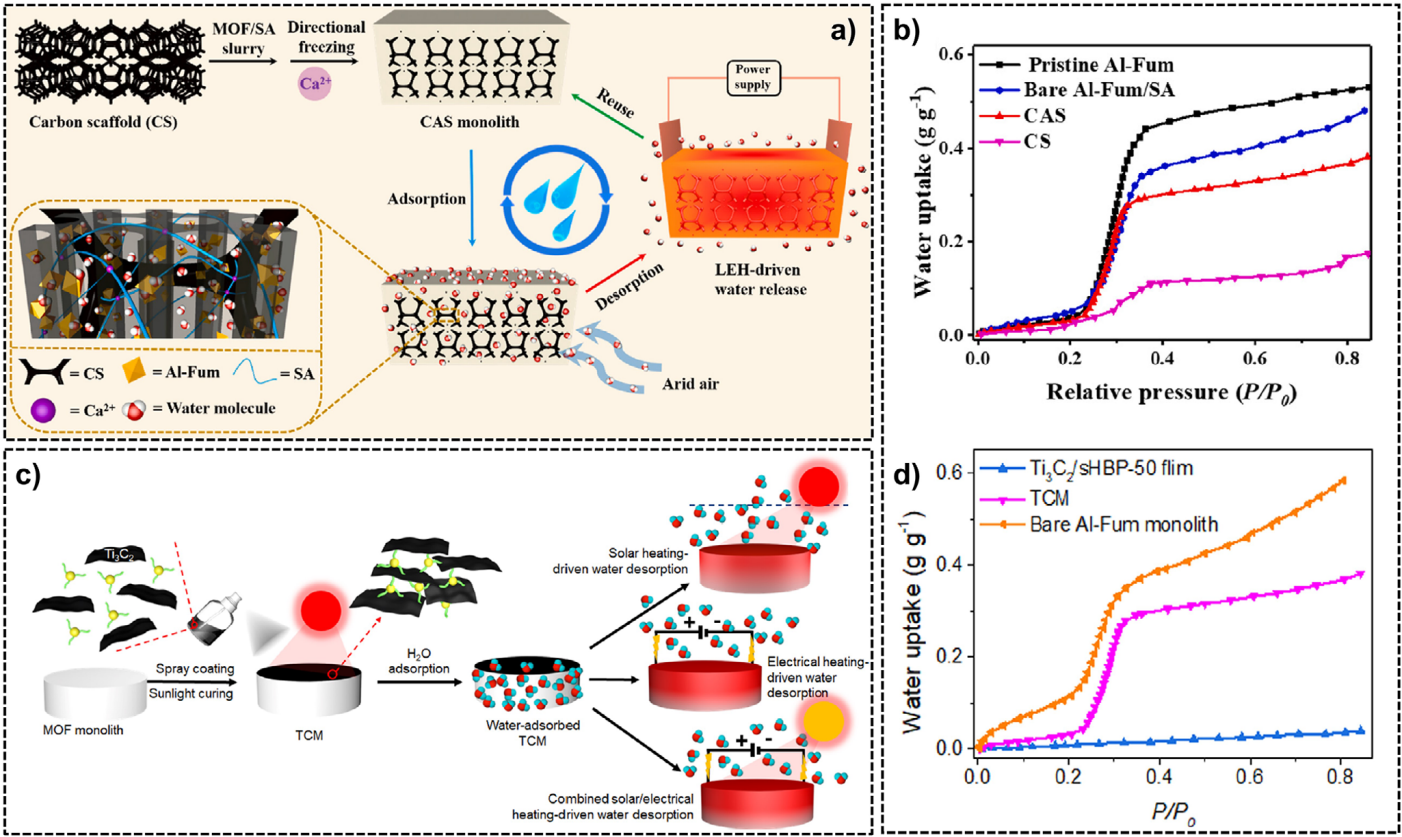
a) Schematic diagram of preparation and AWH performance of Al‐furmarate and SA monolith with carbon scaffold as the conductive grid for LEH‐driven water desorption; b) water uptake isotherm of scaffold Al‐furmarate integrated in SA (CAS) monolith compared with its components (Reproduced with permission.^[^ ^255^ ^]^ Copyright 2022, Elsevier); c) synthesis stages of Ti_3_C_2_/sHBP‐coated MOF monolith (TCM) and its water desorption mechanisms through solar and electrical heating; d) water uptake isotherm of TCM compared with components (Reproduced with permission.^[^ ^257^ ^]^ Copyright 2023, American Chemical Society).

To optimize performance, MOF‐monolith composites are fabricated using direct mixing and in situ growth techniques, ensuring uniform MOF dispersion and maximized active surface area.^[^ ^253^, ^254^ ^]^ One of the recently reported prototypes in terms of MOF‐decorated monolith is the one suggested by Tao et al.^[^ ^255^ ^]^ In their study, they synthesized Al‐fumarate MOF on a cross‐linked sodium alginate (SA) network with the aid of CaCl_2_, integrating this structure onto a carbon scaffold (CAS) characterized by aligned microchannels (**Figure** **12**a). The innovative monolith, referred to as the CAS structure, features a design that triggers water desorption through Joule heating, facilitated by the carbon scaffold's conductivity. This approach not only enhances thermal efficiency but also accelerates the desorption kinetics of water molecules. The aligned channels within the carbon monolith (Figure 12b) alone are insufficient for capturing significant amounts of water. However, after incorporating SA to form the CAS structure, there was a notable improvement in water uptake properties. Their prototype demonstrated impressive water capture capabilities, achieving 1.95 liters per kilogram of MOF‐decorated carbon monolith per day at RH levels ranging from 28% to 38%.

Expanding on this work, Tao et al.^[^ ^256^ ^]^ compared different MOFs—Al‐fumarate, MOF‐801, and MIL‐101(Cr)—decorated onto cross‐linked SA monoliths with Ca^2^⁺ ions as stabilizers. The reported water adsorption capacities were 0.34 g/g (Al‐fumarate at 35% RH), 0.27 g/g (MOF‐801 at 20% RH), and 0.96 g/g (MIL‐101(Cr) at 60% RH). These MOF‐based monoliths were embedded into porous carbon strands (PCS), acting as localized electrical heating elements. Additionally, directional freeze‐drying introduced vertical channels, optimizing mass transfer pathways. To further enhance efficiency, the researchers implemented a fin‐like structure, accelerating water uptake and release cycles. By combining hierarchical structuring, advanced material synthesis, and efficient heating strategies, Tao et al.'s research underscores the potential of MOF‐decorated monoliths in revolutionizing AWH systems. Their work provides a scalable and practical pathway to address global water scarcity, particularly in arid environments, where conventional water sources are limited.

Wu et al.^[^ ^257^ ^]^ introduced a high‐performance AWH system by integrating Ti_3_C_2_ MXene into a ternary composite with Al‐fumarate MOF (TCM), CaCl_2_, and SA, all embedded within silane‐decorated hyperbranched polyglycerol (sHBP), as illustrated in Figure 12c. The Ti_3_C_2_ MXene nanosheets provided dual photothermal and electrothermal functionalities, enhancing water desorption efficiency. By leveraging Ti₃C₂'s heat‐generation ability under sunlight and electrical current, the system achieved a water harvesting rate of 1.8 L kg^−1^ of MOF per day at 35% RH. As shown in Figure 12c, the adsorption isotherm of TCM showed an S‐shaped curve, with an inflection point between 20‐30% RH, similar to bare Al‐Fum MOF. However, TCM exhibited a slightly lower water adsorption capacity (0.31 g/g at 0.35 P/P₀, 25 °C) compared to bare Al‐Fum (0.39 g/g). This reduction was attributed to the Ti_3_C_2_/sHBP coating, which has limited water adsorption capability. Similarly, after 2 h of air exposure at 35% RH and 25 °C, the TCM monolith adsorbed 0.27 g/g of water, slightly lower than 0.32 g/g for Al‐Fum under identical conditions. Despite the minor reduction in adsorption, the rapid desorption enabled by MXene's photothermal and electrothermal effects significantly improved all‐day evaporation efficiency, addressing key challenges in continuous AWH operation.

In a similar study, Wu et al.^[^ ^258^ ^]^ developed a UiO‐66‐NH_2_‐based monolith reinforced with Ti_3_C_2_ MXene (denoted as TUN), where cross‐linked SA acted as the matrix. By incorporating vertical channels, the monolith facilitated faster sorption and desorption cycles, while improving solar penetration and energy absorption. This dual functionality resulted in water capture of 57.8 mL kg^−1^ of MOF in just one hour at 20% RH. However, unlike the S‐shaped sorption behavior typically observed in MOFs, the adsorption isotherm of TUN lacked a distinct inflection point, possibly due to MOF dispersion and accessibility limitations, which may affect performance in certain applications. Both studies highlight the potential of MXene‐integrated MOF‐monoliths in enhancing water harvesting efficiency by combining hierarchical structuring, rapid desorption mechanisms, and advanced nanomaterials.

Wei et al.^[^ ^259^ ^]^ developed a hierarchical porous MOF monolith to address the limitations of powder‐based MOFs in solar‐powered AWH. By fabricating a free‐standing Cu_3_(2,3,6,7,10,11‐hexahydroxytriphenylene)_2_ film with an interconnected porous network, the study enhanced both mass and heat transfer efficiency. The macroporous copper foam substrate facilitates water transport, while the nanowire‐structured MOF layer improves adsorption kinetics and photothermal conversion. The hierarchical structure provides fast water diffusion channels, achieving a water adsorption capacity of 6.13 g m^−2^ and a rapid adsorption rate of 8.16 g m^−2^ h^−1^ at 20% RH. Upon solar exposure, the porous film reaches 75 °C under one sun irradiation, enabling efficient desorption without external energy input. This work highlights how structural engineering at multiple scales enhances water sorption performance, showcasing an approach for designing monolithic MOF‐based AWH systems with integrated adsorption and photothermal properties for use in arid environments. In another study, Luo et al.^[^ ^260^ ^]^ introduced a glass fiber‐supported monolithic MOF adsorbent that integrates dual‐linker Al‐based MOFs (MIL‐160(Al) and Al‐fumarate) for enhanced AWH in low‐humidity conditions. By modifying glass fiber paper (GF) with a chitosan polyelectrolyte layer, the authors achieved a 306.71% increase in MOF loading, significantly improving water uptake efficiency while preventing MOF detachment. The resulting Chitosan‐modified Glass Fiber‐mixed‐MOFs monolith exhibited superior adsorption kinetics, reaching 0.37 g/g at 30% RH and maintaining stable performance over 50 adsorption‐desorption cycles. The hybrid structure improved mixed organic linkers to optimize water sorption isotherms, demonstrating how controlled porosity and surface chemistry tuning can improve mass transport and durability. This study presents a scalable, structurally stable MOF monolith, reinforcing the potential of hybrid frameworks in advancing AWH technologies for arid climates.

**Figure 13:**
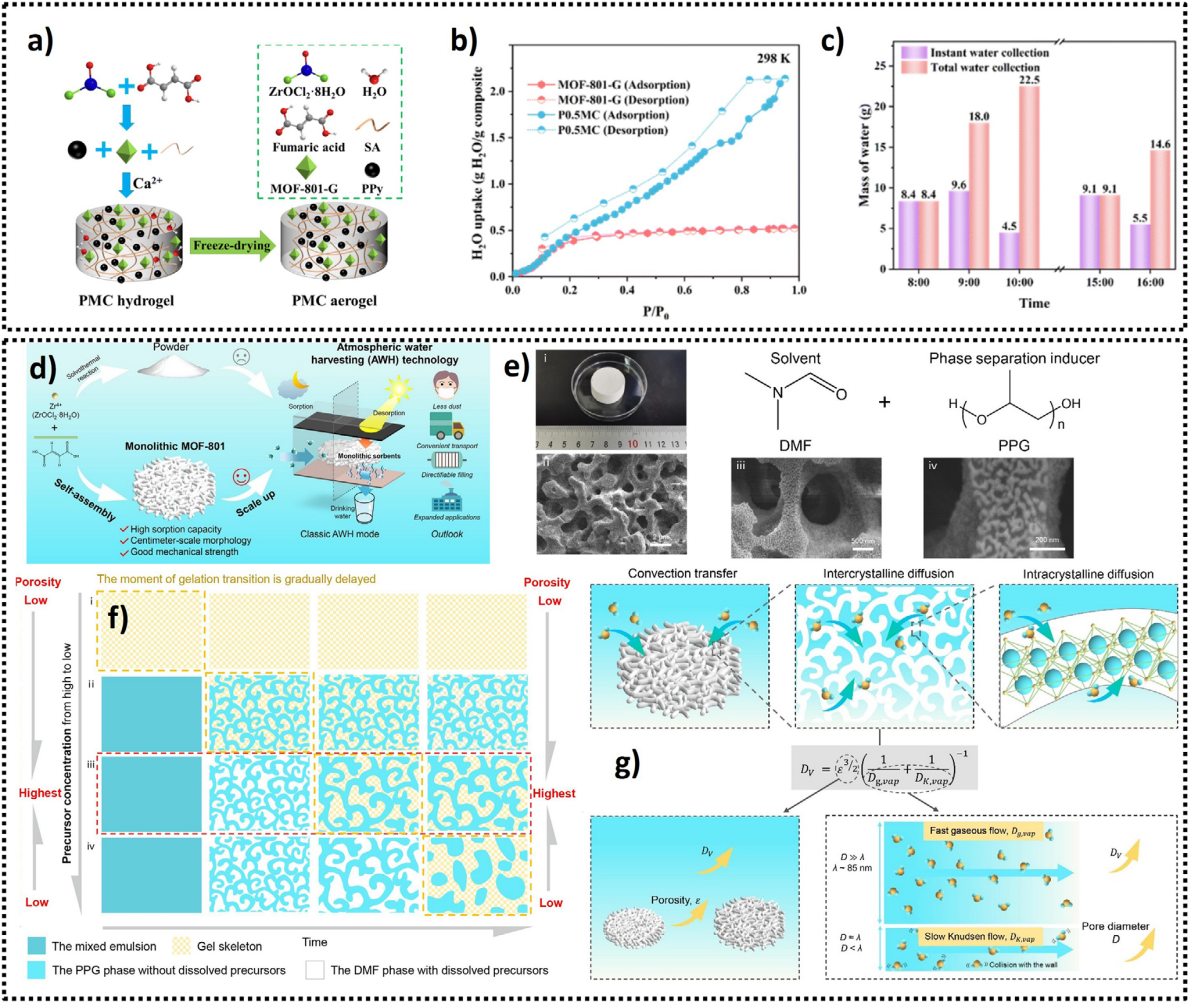
a) Schematic of PMC monolith synthesis via freeze‐drying; b) Water adsorption isotherm of P0.5MC monolith, and c) Real‐world AWH performance (Reproduced with permission.^[^ ^261^ ^]^ Copyright 2024, Royal Society of Chemistry); d) Schematic representation of monolithic MOF‐801 for AWH, highlighting scalability and practical implementation; e) SEM image of the MOF‐801 monolith and the chemical structures of the solvent (N, N‐dimethylformamide (DMF)) and phase separation inducer (poly(propylene glycol (PPG)); f) Controlled porosity tuning based on precursor concentration and gelation time; g) Illustration of mass transfer mechanisms in the MOF monolith system, including convection transfer, intercrystalline diffusion, and intracrystalline diffusion (Reproduced with permission.^[^ ^266^ ^]^ Copyright 2023, Elsevier).

Recently, Liu et al.^[^ ^261^ ^]^ developed a high‐performance AWH system by engineering a porous and hygroscopic monolith (polypyrrole (PPy)/MOF‐801‐G/cross‐linked calcium alginate (CA) or PMC monolith) through the integration of MOF‐801‐G and PPy into a CA matrix, as illustrated in **Figure** **13**a. The freeze‐drying process facilitated the formation of a hierarchical structure, enhancing both water sorption capacity and desorption efficiency. The optimized formulation, P0.5MC, refers to a PMC monolith with 0.5 wt.% PPy and MOF‐801‐G, striking a balance between moisture adsorption, photothermal‐driven desorption, and structural stability. As shown in Figure 13b, the adsorption isotherm of P0.5MC monolith exhibited a Type IV profile, characteristic of microporous materials, with a water uptake of 1.106 g/g at 60% RH—a significant improvement over pristine MOF‐801‐G due to defect engineering and enhanced surface interactions. The material's high surface area and well‐distributed hydrophilic sites contributed to superior water retention, while the photothermal effect of PPy accelerated solar‐driven desorption. In real‐world AWH conditions (Figure 13c), the monolith achieved an impressive total water collection of 22.5 g per cycle, with a daily water harvesting rate of 1.081 kg/kg, meeting WHO drinking water standards. The rapid desorption behavior, driven by solar heating, ensured efficient water release and minimal energy input requirements, making the P0.5MC monolith a promising candidate for scalable AWH applications. However, its performance still relies on solar energy, which may limit its functionality under low‐light or nocturnal conditions.

Traditional passive AWH systems rely on solar‐driven desorption, limiting on‐demand water release due to fluctuations in solar availability. To address this, it is possible to incorporate polymers with lower critical solution temperature (LCST) behavior, which enable autonomous water release due to temperature‐induced phase transitions. This approach allows confined water molecules to detach from polymer chains and coalesce into liquid upon reaching the LCST, ensuring continuous sorption‐desorption cycles without external energy input. For example, Yilmaz et al.^[^ ^262^ ^]^ d applied this technique to hydrogels, integrating MIL‐101(Cr) MOF within a PNIPAM‐based network to achieve efficient, spontaneous water release above 32 °C, independent of solar intensity. Similarly, some studies have explored this technique for fog harvesting and other water collection applications,^[^ ^263^, ^264^, ^265^ ^]^ suggesting a promising future direction for improving AWH performance.

He et al.^[^ ^266^ ^]^ developed a hierarchically porous MOF‐801 monolith to enhance the efficiency of atmospheric AWH by overcoming mass transfer limitations and handling challenges associated with powder‐based MOFs (Figure 13d). The monolith was fabricated using a sol‐gel phase separation method followed by acid‐heat post‐treatment, allowing precise control over porosity. A dimethylformamide (DMF) and poly(propylene glycol) (PPG) system was introduced to induce phase separation, where DMF served as the solvent, dissolving metal salts and organic ligands, while PPG acted as the phase‐separation agent (Figure 13e). Since the MOF precursors could not dissolve in PPG, a biphasic emulsion was formed, initially homogeneous under stirring but gradually undergoing phase separation during gelation. Upon gelation, the DMF‐rich phase solidified into a continuous MOF network, while PPG created interconnected macropores. The resulting monolithic MOF‐801 maintained its shape after drying, preserving a highly porous structure (Figure 13e‐i–iv). By optimizing gelation timing and precursor concentration, the study demonstrated that porosity and pore size could be precisely tuned, as illustrated in Figure 13f. A precursor concentration that delayed gelation transition to coincide with phase separation yielded the highest porosity (59.7%), whereas excessive precursor content led to compact structures with reduced macroporosity.

The hierarchical porous network played a crucial role in vapor transport and sorption efficiency. The monolith exhibited a high micropore volume (0.234 cm^3^ g^−1^) and surface area (977.8 m^2^ g^−1^), significantly improving adsorption capacity. Compared to MOF‐801 powders and compressed tablets, the monolithic MOF‐801 exhibited 1.2‐ and 1.4‐fold higher water uptake at 30% RH, respectively, highlighting the impact of structural engineering on vapor adsorption kinetics. As shown in Figure 13g, the study further analyzed water vapor diffusion mechanisms, demonstrating that mass transport occurred in three stages: I) convection‐driven external diffusion, II) intercrystalline diffusion through macropores, and III) intracrystalline diffusion into micropores. Among these, intercrystalline diffusion played a dominant role, as it depended on porosity and pore diameter. The monolith's macropore size (≈650 nm) was much larger than the mean free path of water molecules (85 nm), allowing for rapid gaseous diffusion and efficient transport across the monolith. With porosity 2.8 times higher than that of MOF‐801 tablets, the monolithic MOF‐801 demonstrated enhanced adsorption even after scale‐up. By integrating macro‐microporous structuring with scalable monolithic shaping, this study provides a feasible pathway for high‐performance MOF‐based atmospheric water harvesting, offering an alternative to powder‐based materials with improved transport dynamics and practical applicability.

Overall, MOF‐decorated monoliths with hierarchical porosity offer a highly effective solution for improving water capture, stability, and transport efficiency in AWH. Their optimized structure prevents pore collapse, enhances mass and heat transfer, and accelerates adsorption‐desorption cycles, making them ideal for scalable applications. While these materials address small‐scale challenges, the next section focuses on macro‐scale AWH system design, including scalability, infrastructure integration, and economic feasibility.

## 6 Macroscale Design: AWH Devices

The ultimate purpose of considering AWH at different scales is to provide a platform for optimizing the parameters involved to expand their utility on the macro scale. Generally, a water harvester is comprised of a sorbent bed, a condenser, and a section for collecting the produced water.^[^ ^267^ ^]^ Based on the sorption‐desorption cycle, researchers have proposed multiple practical scenarios, which can be categorized into three main groups: monocyclic, multicyclic, and continuous harvesters. In this context, several key considerations must be addressed for the optimized design of next‐generation water harvesting devices.

The first and simplest type of atmospheric water harvesters are monocyclic devices. These devices operate with a single sorption‐desorption cycle over a 24‐hour period. In this approach, sorption occurs at night, followed by desorption during the day, driven by solar radiation.^[^ ^268^ ^]^

To increase water yield, multicyclic setups have been introduced, which undergo multiple sorption‐desorption cycles throughout the day. Therefore, the duration of each cycle and, consequently, sorption/desorption kinetics, influence the system's productivity.^[^ ^269^ ^]^ Almassad et al.^[^ ^270^ ^]^ fabricated a multicyclic device with the aid of MOF‐801 trays as the sorbent material, delivering 3.5 L_H2O_/kg_MOF_ per day. One of its outstanding features is an adaptive mode of the device evaluating the external ambient conditions (RH and temperature) to adjust adsorption and desorption phases aimed at the optimized condition for maximum output.

All the previous discussions sowed the seeds for the optimal design of a continuous system.^[^ ^271^ ^]^ In continuous systems, the use of LCST‐type polymers is highly recommended due to their thermally responsive nature, which allows sorbent to continuously produce water without relying on solar radiation or external energy sources for evaporation and condensation. Several devices have been designed by different research groups, and their performance, along with the conditions under which they capture and release water, are reviewed in **Table** **5**.

**Table 5:** Summary of recent fabricated AWH devices.

Orbent components^[^ ^272^ ^]^	Overall water collection	RH [%]	Desorption driving force	Sorption‐desorption cycle time	Mode of operation	WHO standard of water quality	Remark	Refs.
ZIF‐67, Carbonized PAN, Co nanoparticles	4.5 L_H2O_/kg.day	30	Localized magnetic induction heating (LMIH)	20 min	Multicyclic	Pass	–	[272]
MIL‐101‐Cr (HF), P(VdF‐HFP)	3.2 mL/g_MIL‐101‐Cr(HF)_.day	50‐65	Radiative condenser	–	Multicyclic	–	–	[142]
MOF‐801	3.5 L_H2O_/kg_MOF_.day	17‐32	Electric heater (3‐7 kwh L^−1^)	90 min	Multicyclic	Pass	Tray‐based design	[270]
MOF‐303	1.3 L/kg_MOF_.day	32	Electrical heating by solar module	460 min	Multicyclic	–		[144]
MOF‐303, Graphite, Porous Ni foam	210 g/ kg_MOF_.day 258 g/ kg_MOF_.day	26 86	Sunlight heating	24 h	Monocyclic	–	Cartilage‐based passive design	[273]
AQSOA Z01 Zeolite	0.77 L/m^2^.day	35	Sunlight heating	24 h	Monocyclic	Failed	Dual‐stage design for recycling heat of condensation	[274]
Al‐fumarate, MIL‐88A, PANI, Chitosan‐modified glass fiber	119 g/g_MOF_.day	–	Sunlight heating	60 min	Multicyclic	Pass	–	[152]
LiCl, Nano carbon Hallow capsule	1.6 kg/kg_sorbent_	60	Sunlight heating	3.5 h	Continuous	Pass	1.25 g harvested water in 75% RH after 4 hours	[275]
SA, CaCl_2_, graphite	2.48 – 3.3 L/kg_sorbent_.day	30‐50	Heat exchanger	3h	Continuous	–	Radial fin decorated cylinder for heat & mass transfer boosting	[276]
MIL‐101(Cr)	7.7‐22.8 L/kg_MOF_.day	20‐80	Heat pump & air cooling	–	Continuous		–	[277]

## 7 Conclusion and Future Prospects

This review discussed the methods for effective AWH by integrating MOF species into macroscopic porous constructs to address water scarcity. To this end, we show the promise of combining MOFs with porous macroscopic frameworks, such as monoliths, for creating hierarchical structures with improved structural integrity. MOFs, with their highly porous nanoscale structures and versatile chemical tunability, offer significant potential for AWH, particularly in arid environments. However, their limited large‐scale processability and mechanical stability hinder their practical deployment. By hybridizing MOFs with monoliths, it is possible to engineer hierarchical porous materials that enhance water uptake, structural integrity, and scalability.

Combining MOFs with monoliths makes their pores better accessible for water harvesting. MOFs have a limited capacity for water uptake and suffer from assembly issues at the macro‐scale. Monoliths, though slow in terms of sorption ability, complement MOFs' shortcomings in cases of shape‐ability and water uptake capacity. Hierarchical porous materials combining MOFs and monoliths offer optimized properties, processability, and functionality, addressing the limitations of neat MOFs or traditional monoliths. This review covered the fundamentals of sorption and desorption with a hierarchical perspective on porous materials for AWH. We discussed recent advancements in AWH technologies, focusing on material design and optimization, highlighting the significant strides made toward operational efficiency and effectiveness. At the core of these advancements is a deep understanding of the sorption mechanisms, particularly the interactions between water and sorbent molecules. Such knowledge is crucial for elucidating the structure‐property relationships of sorbents, paving the way for the development of materials.

The future of porous materials lies in their continued development and optimization for specific applications. Researchers should explore the possibilities of hierarchical porous structures by combining different porous materials and integrating multiscale porosity. This approach opens new avenues for a broad range of applications, especially AWH. Addressing these areas can advance the field of MOF‐based monoliths and unlock their full potential in various technological domains. We recommend that future studies focus on the areas set out below.

### 7.1 Nature‐Inspired Nanotechnological Solutions

Millions of years of evolution have equipped species with remarkable adaptations for survival, making nature an attractive model for studying how living entities harvest water from the atmosphere. This opens up a wide range of opportunities by mimicking the technical principles of nature and applying them to the design of nanotechnological products. For example, MOF monoliths can be engineered to mimic the structural designs and material characteristics of desert beetles, spider webs, and cacti—all of which harvest and collect water from the atmosphere at different humidity levels. By integrating these natural strategies, MOF monoliths can be optimized for enhanced water capture and release, offering a promising approach for efficient AWH.

### 7.2 Exploration of Novel Materials

Future research should focus on identifying and developing novel MOFs and monoliths with enhanced stability and durability when in contact with water. This could involve synthesizing new organic ligands, exploring different metal combinations, and investigating alternative fabrication methods. Additionally, incorporating hygroscopic, nature‐derived materials such as cellulose nanomaterials as the core of monoliths in hybrid with MOFs could lead to promising green water harvesting technologies. The use of thermoresponsive materials, such as Poly(N‐isopropylacrylamide) (PNIPAM), offers an effective approach to adsorb and desorb water by changing the material's character from hydrophilic to hydrophobic when heated beyond a specific temperature. Looking ahead, AI and machine learning hold significant potential to advance the study and development of new materials. These technologies can streamline the discovery and optimization processes by predicting material behaviors and properties based on vast datasets.

### 7.3 Sustainable Bio‐Based AWH Materials

The emphasis on developing sustainable bio‐based materials for AWH reflects a growing recognition of the environmental and economic challenges associated with petroleum‐based materials. Bio‐based materials, derived from renewable biological resources, offer a compelling alternative due to their potential for sustainability, biodegradability, and reduced environmental impact. In the context of AWH, the shift toward bio‐based materials is not merely a matter of environmental responsibility but also a strategic approach to enhancing the sustainability and efficiency of water harvesting technologies. These materials, such as cellulose, chitosan, and starch, can be designed to possess inherent properties such as high water affinity, selectivity, and favorable sorption–desorption dynamics, making them ideally suited for water capture and release in diverse environmental conditions.

### 7.4 Precise Porosity Engineering and Structuring

Another crucial aspect of water harvesting systems is the ability to control the porosity of manufactured water harvesters across different scales to maximize water uptake at varying humidity levels. Additionally, incorporating specific structural features, such as those observed in desert beetles, or implementing a gradient of hydrophobicity combined with tailored surface features, could serve as effective tools to accelerate water harvesting and ensure efficient release. Heterogeneous surfaces with a combination of hydrophilic and hydrophobic sites can also provide a versatile solution for optimizing water harvesting and release under sunlight.

### 7.5 Stability and Durability

Enhancing the stability and durability of MOF‐based monoliths is a critical research direction. Exploring strategies to improve resistance to degradation, moisture, and chemical exposure is essential for ensuring their long‐term performance and practical application. It is worth mentioning that machine learning models can predict the long‐term stability and durability of MOF monoliths, guiding the development of materials with improved resistance to degradation, moisture, and chemical exposure.

### 7.6 Cost and Scalability

Future research should focus on developing scalable and cost‐effective methods for producing MOF‐monoliths. High production costs stem from the use of expensive organic linkers and metal precursors, as well as energy‐intensive solvothermal synthesis processes, which pose significant challenges for large‐scale applications.^[^ ^278^ ^]^

To address these limitations, researchers are exploring cost‐effective synthesis approaches, such as solvent‐free synthesis and the use of water as a solvent.^[^ ^279^ ^]^ However, despite these advancements, the economic feasibility of MOF‐based AWH devices remains a concern. To enhance scalability and commercial viability, innovative fabrication techniques—including continuous flow synthesis, template‐assisted fabrication, and 3D printing—should be further investigated for large‐scale production.

### 7.7 Advanced Characterization Techniques

Developing advanced characterization techniques is crucial for understanding the structure‐property relationships in MOF monoliths. High‐resolution imaging, spectroscopic analysis, and in situ characterization methods can provide valuable insights into these materials' properties and behaviors. In particular, understanding the interactions between MOFs and other materials remains challenging, and accurately measuring the loading of MOFs often lacks clarity. Addressing these issues is essential to improving the reliability and applicability of these materials.

### 7.8 Application‐Specific Design and Multifunctional Systems

Tailoring MOF‐monoliths' properties for specific applications (or to particular environments) will be an exciting research avenue. This could include design strategies that optimize porosity, surface chemistry, and mechanical strength for a targeted temperature range or RH. The considered strategy could incorporate nanoparticles, polymers, or biomass to enhance specific functionalities like sorption and desorption.

In summary, the study of porous materials, inspired by nature and guided by scientific ingenuity, holds immense potential for addressing current challenges and unlocking new possibilities in various industries. By harnessing the unique properties of porous materials and exploring the synergistic effects of hierarchical structures, scientists can continue advancing the development of functional materials to benefit society and contribute to a sustainable future.

## Conflict of Interest

The authors declare no conflict of interest.
